# Dietary assessment in UK Biobank: an evaluation of the performance of the touchscreen dietary questionnaire

**DOI:** 10.1017/jns.2017.66

**Published:** 2018-02-01

**Authors:** Kathryn E. Bradbury, Heather J. Young, Wenji Guo, Timothy J. Key

**Affiliations:** Cancer Epidemiology Unit, Nuffield Department of Population Health, University of Oxford, Oxford, UK

**Keywords:** UK Biobank, Dietary assessment, Reproducibility, Touchscreen questionnaires, NDNS, UK National Diet and Nutrition Survey

## Abstract

UK Biobank is an open access prospective cohort of 500 000 men and women. Information on the frequency of consumption of main foods was collected at recruitment with a touchscreen questionnaire; prior to examining the associations between diet and disease, it is essential to evaluate the performance of the dietary touchscreen questionnaire. The objectives of the present paper are to: describe the repeatability of the touchscreen questionnaire in participants (*n* 20 348) who repeated the assessment centre visit approximately 4 years after recruitment, and compare the dietary touchscreen variables with mean intakes from participants (*n* 140 080) who completed at least one of the four web-based 24-h dietary assessments post-recruitment. For fish and meat items, 90 % or more of participants reported the same or adjacent category of intake at the repeat assessment visit; for vegetables and fruit, and for a derived partial fibre score (in fifths), 70 % or more of participants were classified into the same or adjacent category of intake (κ_weighted_ > 0·50 for all). Participants were also categorised based on their responses to the dietary touchscreen questionnaire at recruitment, and within each category the group mean intake of the same food group or nutrient from participants who had completed at least one web-based 24-h dietary assessment was calculated. The comparison showed that the dietary touchscreen variables, available on the full cohort, reliably rank participants according to intakes of the main food groups.

UK Biobank is a prospective cohort of half a million men and women from across the UK. Information on a broad range of exposures, including diet, was collected from the participants at an assessment centre, and linkage to cancer and death registries, as well as other medical records, enables many hypotheses to be examined^(^^1^^)^. The UK Biobank dataset is an open access resource; any *bona fide* researcher can apply to use the data for health-related research that is in the public interest^(^^1^^)^.

At recruitment, the touchscreen questionnaire used in UK Biobank asked twenty-nine questions about diet, most of which gathered information about the average frequency of consumption of main foods and food groups over the past year. Prior to using the data from the touchscreen questionnaire in diet–disease analyses, it is important to examine the reproducibility of the dietary questions. Future studies in UK Biobank may rank participants according to dietary intake from the touchscreen questionnaire and assess relative risk of disease across categories of intakes; misclassification in the ranking of participants according to dietary intakes will be expected to underestimate associations between diet and disease risk^(^^2^^)^. Using a subsample of approximately 20 000 participants who completed a repeat of the assessment centre visit^(^^3^^)^, about 4 years after recruitment, enables an examination of the combination of the variation in response to the questionnaire as well as true changes in intake over time, both of which contribute to misclassification of long-term dietary intakes^(^^4^^)^.

The agreement between the touchscreen questionnaire, which asked about frequency of consumption, and a more detailed dietary assessment method which gathers information on actual amounts of food consumed, can be used to further evaluate the touchscreen dietary data. A web-based 24-h dietary assessment tool^(^^5^^)^ was also used in UK Biobank to gather additional information on dietary intakes; over 200 000 participants completed at least one 24-h dietary assessment, and the mean intakes from the 24-h dietary assessments can be used for this purpose.

The objectives of the present paper are to describe the reproducibility of the touchscreen questions, using the subsample of participants who repeated the assessment centre visit, and to examine the agreement between the dietary touchscreen variables and the group mean intakes from the web-based 24-h dietary assessments conducted on a large subsample of participants.

## Methods

### UK Biobank

UK Biobank is a prospective cohort of half a million middle-aged men and women recruited from the UK in 2006 (pilot phase) and 2007–2010 (main phase). People aged 40–69 years who lived within reasonable travelling distance (25 km) of one of the twenty-two assessment centres in England, Scotland and Wales were identified from National Health Service patient registers and invited to attend an assessment centre. Permission for access to patient records for recruitment was approved by the Patient Information Advisory Group (subsequently replaced by the National Information Governance Board for Health and Social Care) in England and Wales, and the Community Health Index Advisory Group in Scotland. At the UK Biobank assessment centres, a touchscreen questionnaire was used to collect information on sociodemographic characteristics, diet and other lifestyle exposures, general health, and medical history. Physical measurements were also taken and participants provided blood and urine samples. Participants are followed up via linkage to cancer and death registries, as well as other health records^(^^1^^)^.

This study was conducted according to the guidelines laid down in the Declaration of Helsinki and all procedures involving human subjects/patients were approved by the North West Multi-centre Research Ethics Committee. At the touchscreen station, all participants gave informed consent to participate in UK Biobank and be followed up, using a signature capture device. The UK Biobank protocol is available online (http://www.ukbiobank.ac.uk/wp-content/uploads/2011/11/UK-Biobank-Protocol.pdf). The touchscreen questionnaire and other resources are available on the UK Biobank website (http://www.ukbiobank.ac.uk/resources/).

### Dietary assessment

#### Touchscreen questionnaire

The touchscreen questionnaire used in the main study contained twenty-nine questions about diet and eighteen questions about alcohol. The touchscreen questionnaire asked about the frequency of consumption over the past year of the following food groups: cooked vegetables, salad/raw vegetables, fresh fruit, dried fruit, oily fish, other fish, processed meats, poultry, beef, lamb, pork, cheese, salt added to food, tea, water, as well as questions on the type of milk most commonly consumed, type of spread most commonly consumed, number of slices and type of bread most commonly consumed, number of bowls and type of breakfast cereal most commonly consumed, cups of coffee and type most commonly consumed, as well as questions on the avoidance of specific foods and food groups (eggs, dairy products, wheat, sugar), age last ate meat (for participants who reported never consuming processed meats, poultry, beef, lamb or pork), temperature preference of hot drinks, changes in diet in the past 5 years, and variation in diet. Four of the dietary questions used in the pilot study were altered slightly for the main phase: these were the questions on avoiding specific foods and food groups; spread type; bread type; and variation in diet. A total of 3776 participants completed only the pilot version of the touchscreen; for analyses on these questions the participants answering only the pilot version were excluded. Details of the possible answers for each dietary touchscreen question are given in the Supplementary Methods^(^^6^^,^^7^^)^. We also generated a partial fibre score from the touchscreen questionnaire using the questions on fresh fruit, dried fruit, raw vegetables, cooked vegetables, bread type and bread intake, and breakfast cereal type and breakfast cereal intake. Further detail on how we generated the partial score is given in the Supplementary Methods and Supplementary Table S1.

#### Web-based 24-h dietary assessments

In early 2009, the main study protocol was modified to include a number of enhancements to the assessment centre visit^(^^8^^)^. These enhancements included the Oxford WebQ, a web-based 24-h dietary assessment tool, which asks about the consumption of up to 206 types of foods and thirty-two types of drinks during the previous 24 h. The mean daily intakes of nutrients were calculated by multiplying the frequency of consumption of each food or drink by a standard portion size and the nutrient composition of that particular item. The web-based 24-h dietary assessment has been compared with an interviewer-administered 24-h recall completed on the same day, with Spearman's correlation coefficients for the majority of nutrients calculated from the WebQ ranging between 0·5 and 0·9 (mean of 0·6)^(^^5^^)^. Participants who were recruited between April 2009 and September 2010 completed the 24-h dietary assessment at the assessment centre. In addition, after the recruitment period closed, an email was also sent out every 3–4 months a total of four times between February 2011 and June 2012 (online cycle 1, February 2011 to April 2011; online cycle 2, June 2011 to September 2011; online cycle 3, October 2011 to December 2011; online cycle 4, April 2012 to June 2012) to participants who had provided an email address at recruitment, inviting them to complete the Oxford WebQ online using their own computer. The email invitations were sent on variable days of the week, and participants were given 3 d to complete it for cycles 1 and 2, and this was extended to 14 d for cycles 3 and 4, after which the link expired. For all analyses, we excluded 24-h dietary assessments where the energy intakes were greater than 20 000 kJ for men (1758 records from a total of 203 955 (0·86 %)), and 18 000 kJ for women (1736 records from a total of 254 798 (0·68 %)). In a sensitivity analysis, we also excluded 24-h dietary assessments where participants specified that their diet for that day was not typical because of fasting or illness; this did not have a large effect on the results, so all results are reported with these participants included.

#### Repeatability of the touchscreen questionnaire

Approximately 20 000 participants who resided in the area surrounding UK Biobank's coordinating centre in Stockport undertook a full repeat of the assessment centre visit, between August 2012 and June 2013, approximately 4 years after recruitment^(^^3^^)^.

To assess the long-term repeatability of the dietary questions on the touchscreen questionnaire, as well as the new partial fibre score, we used the subsample of participants who had completed the repeat assessment centre visit and examined the agreement between participants’ responses to the dietary questions on the touchscreen questionnaire completed at baseline and the repeat visit. For this analysis, questions where the possible responses were categorical, i.e. questions on fish, meat, cheese, types of milk, spread, bread, cereal, salt added to food, temperature of hot drinks, major changes to diet, and variation in diet, we cross-tabulated the answers as recorded. For questions that used direct entry responses, we truncated or collapsed answers into categories to enable cross-tabulation as follows: for servings of fruit and vegetables we used 0, 1, 2, 3, 4, ≥5; for the derived partial fibre score we categorised participants into fifths based on the whole cohort. For age last ate meat we used 0–10, 11–20, 21–30, 31–40, 41–50, 51–60, ≥61 years; for slices of bread we used 1–5, 6–10, 11–15, 10–20, 21–25, 26–30, ≥31; for bowls of breakfast cereal we used 0, 1, 2, 3, 4, 5, 6, 7, ≥8, for cups of tea and coffee and glasses of water we used 0, 1, 2, 3, 4, 5, ≥6. For all questions, participants selecting ‘do not know’, ‘prefer not to answer’ or ‘less than one’ were assigned to separate categories, except for number of bread slices where ‘less than one’ was combined with ‘0’ because of very low numbers for both of these groups. For the question on foods avoided we created binary variables for each food item, e.g. consumers/non-consumers of dairy products.

After excluding participants who answered ‘do not know’ or ‘prefer not to answer’ at either baseline or the repeat visit, we also assessed agreement using the κ coefficient. Bootstrapping with 10 000 replications was used to calculate CI around the κ coefficient. In a separate analysis, we also further excluded participants who reported, at the repeat visit, making a major change to their diet in the past 5 years. We also examined κ coefficients by sex, age (<55 years, ≥55 years) and BMI (<25 kg/m^2^, ≥25 kg/m^2^). For most of the dietary touchscreen questions, the categories of responses to the dietary questions were ordinal, ranging from least frequently eaten to most frequently eaten; therefore for these questions the κ coefficient with quadratic weighting was used, which is equivalent to the intra-class correlation coefficient and allows for the fact that a change from category 1 to category 2 reflects closer agreement over time than, for example, a change from category 1 to category 4^(^^9^^)^. κ Values >0·80 indicate excellent agreement, values between 0·61–0·80 substantial agreement, 0·41–0·60 moderate agreement, 0·21–0·40 fair agreement, and ≤0·20 poor agreement^(^^10^^)^. For questions where the responses were not ordinal, e.g. bread or spread type mainly used, only the percentage in the same category is given, κ values were not calculated.

#### Agreement between the intakes of foods and food groups and the partial fibre score from the touchscreen dietary questions and group mean intakes from the 24-h dietary assessment

We created new variables for the weight (in g) from the 24-h dietary assessments of the food groups that were included in the touchscreen questionnaire: total vegetables; total fresh fruit; total dried fruit; oily fish; other fish; processed meat; poultry; beef; lamb; pork; cheese; tea; caffeinated coffee; decaffeinated coffee; water; white sliced bread; granary brown or mixed flours sliced bread; wholemeal sliced bread; bran cereal; wholewheat cereals; porridge; muesli; and plain cereals, sweetened oat crunch-type cereals, other sweetened cereals, and other cereals. This was done by using the steering file of the Oxford WebQ which contains the serving size of each food item listed in the 24-h dietary assessment, in g (data not shown). To estimate daily intake, the serving size in g was multiplied by the frequency reported in the 24-h dietary assessment. The top frequency category was open ended and differed by food group; these were coded so that 3+ = 3, 4+ = 4, 5+ = 5, 6+ = 6. Less than one was coded as 0·5. Dietary variables from the 24-h dietary assessments were chosen to match the touchscreen food groups as closely as possible; details of the individual dietary variables from the 24-h dietary assessment that formed each food group are given in Supplementary Table S2.

For the purposes of comparing the touchscreen dietary variables with the 24-h dietary assessments, we grouped participants into categories for each food group based on the touchscreen questionnaire (for the main food groups of meat, fruit and vegetables we typically used four categories per food group), and these were compared with the 24-h dietary assessments in two ways. Firstly, within each category we calculated the mean intake (in g) of the same food group from participants who completed the 24-h dietary assessment tool at the assessment centre; this first analysis shows how well the touchscreen and 24-h dietary assessment tool agree when they are completed on the same day. Secondly, in each category we calculated the group mean intake of the corresponding food group from 24-h dietary assessments from participants who had completed one or more online 24-h dietary assessments. For participants who completed more than one online 24-h dietary assessments, we first averaged the values from all of their completed online dietary assessments. In this second analysis, we excluded the 24-h dietary assessments that were completed at the assessment centre on the same day as the touchscreen questionnaire; we used only the online 24-h dietary assessments because the aim was to take into account both change over time and variation in day-to day intakes, and therefore we wanted a lag time of at least a few months between the touchscreen questionnaire and the 24-h dietary assessment. To rank the participants by weekly red meat consumption based on the touchscreen, we summed the frequencies for beef, pork and lamb/mutton, using the following coding: ‘never’ = 0, ‘less than once per week’ = 0·5, ‘once per week’ = 1, ‘2–4 times per week’ = 3, ‘5–6 times per week’ = 5·5, ‘once or more daily’ = 7. The same approach was used for total meat consumption based on the touchscreen, which was the sum of processed meat, poultry, beef, pork and lamb/mutton.

## Results

**Fig. 1.**
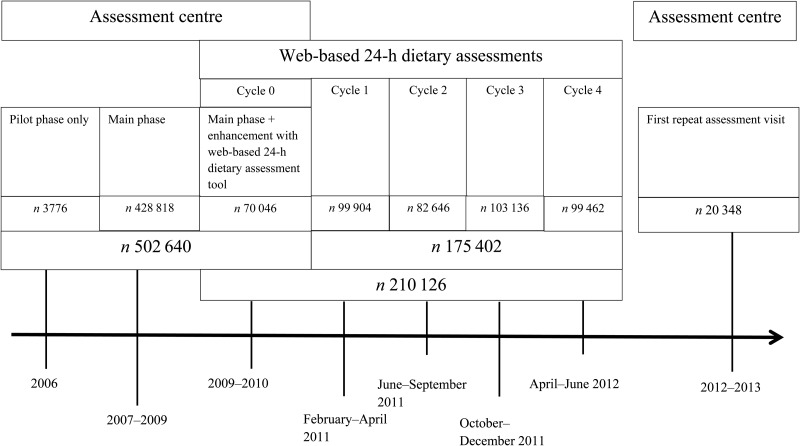
UK Biobank participant flow showing numbers of people who participated in the assessment centre visit at recruitment, the web-based 24-h dietary assessments, and the repeat assessment centre visit.

The initial dataset for the present study consisted of 502 640 participants who completed the touchscreen questionnaire at the recruitment visit. Of the participants, 20 348 repeated the touchscreen questions at the repeated assessment centre visit; the median time between administrations was 4·4 (25th–75th percentile 3·7–5·0) years. In total, 210 128 participants completed at least one 24-h dietary assessment with plausible energy intakes and 126 096 completed at least two 24-h dietary assessments; 70 046 participants completed a 24-h dietary assessment at the recruitment centre, and about half of these participants (*n* 35 322) also completed one or more online 24-h dietary assessments (Fig. 1 and Table 1). Basic participant characteristics are shown for the whole cohort, the subsample that completed a repeat assessment centre visit, and the subsample that completed at least one 24-h dietary assessment in Table 2. Participants who completed the repeat assessment centre visit or at least one 24-h dietary assessment were more likely to have a university degree or vocational qualification and slightly less likely to smoke, compared with the full cohort.

**Table 1.** Number of participants who completed the web-based 24-h dietary assessment

	Total number of 24-h dietary assessments					
	1	2	3	4	5	Total
Recruitment (*n*)*	34 724	11 404	9693	8620	5605	70 046
Online only (*n*)†	49 307	36 856	32 609	21 308	–	140 080
Total (*n*)	84 031	48 260	42 302	29 928	5605	210 126

* These are the participants who completed a 24-h dietary assessment at the assessment centre at recruitment. Some participants also completed online 24-h dietary assessments.

† These are the participants who did not complete a 24-h dietary assessment at the assessment centre at recruitment. They completed one or more online 24-h dietary assessments.

**Table 2.** Participant characteristics in the full UK Biobank cohort and in the subsamples who completed the repeat assessment and the 24-h dietary assessments (Numbers of subjects and percentages; mean values and standard deviations)

	All participants		Participants who completed repeat assessment centre visit		Participants who completed at least one 24-h dietary assessment		Participants who completed a 24-h dietary assessment at recruitment (cycle 0)*		Participants who only completed a 24-h dietary assessment online (cycles 1–4)†	
Characteristic	%	*n*	%	*n*	%	*n*	%	*n*	%	*n*
Subjects (*n*)	502 640		20 348		210 126		70 046		140 080	
Men	45·6	229 174	48·9	9940	44·9	94 304	44·7	31 281	45·0	63 023
Age (years)										
Mean	56·5		57·1		56·1		56·2		56·0	
sd	8·1		7·4		7·9		8·2		7·8	
University or college degree or vocational qualification	59·0	296 655	70·9	14 429	69·6	146 302	66·0	46 250	71·4	100 052
Current smokers	10·6	52 989	6·3	1283	7·8	16 444	8·4	5876	7·6	10 568
Daily or almost daily consume alcohol	20·3	101 790	22·5	4568	22·8	47 980	21·9	15 355	23·3	32 625
High physical activity level‡	17·5	87 739	16·2	3297	16·8	35 299	18·6	13 031	15·9	22 268

* Participants completed cycle 0 of the 24-h dietary assessment and may also have completed any of cycles 1–4.

† Participants completed at least one of cycle 1–4 of the 24-h dietary assessment, but did not complete cycle 0.

‡ High physical activity level is defined as >50 excess metabolic-equivalent hours per week.

### Repeatability of the touchscreen questionnaire

Table 3 shows the responses to the fruit and vegetable questions and the categorisation of participants into fifths based on the new partial fibre score, from the touchscreen questionnaire completed at baseline and at the repeat visit, for the subsample of participants who completed a repeat visit. Table 4 shows the results for the meat and fish questions. Table 5 shows the agreement and κ coefficient with quadratric weighting (κ_weighted_) for these questions. Generally there was good agreement between reported consumption at recruitment and at the repeat assessment centre visit, approximately 4 years later. After excluding participants who selected ‘prefer not to answer’ or ‘do not know’ at either the recruitment or repeat assessment centre visit, the percentage of participants who were classified into the same or adjacent categories (same category only, total number of categories) was 82 % (42 %, seven categories) for cooked vegetables, 72 % (37 %, seven categories) for raw vegetables, 82 % (43 %, seven categories) for fresh fruit, 72 % (51 %, seven categories) for dried fruit, and above 95 % (above 55 %, six categories for each item) for all fish and meat items, except for processed meat which was 90 % (52 %, six categories). The weighted κ coefficient showed substantial agreement for fresh fruit, oily fish, processed meat, poultry, beef, and lamb, and moderate agreement for cooked vegetables, raw/salad vegetables, dried fruit, partial fibre score, other types of fish (non-oily) and pork. After excluding participants who reported that they made a major change to their diet in the past 5 years at the repeat visit, the weighted κ coefficient increased slightly for all items, and fibre and pork now showed substantial agreement. The κ coefficients were similar for men and women, and younger and older participants. Participants with a BMI < 25 kg/m^2^ had higher κ coefficients than participants with BMI ≥ 25 kg/m^2^ (Supplementary Table S3). The agreement for the other touchscreen dietary variables is shown in Supplementary Table S4.

**Table 3.** Reported daily consumption of fruit and vegetables among 20 348 participants who answered the dietary touchscreen questionnaire about 4 years apart*

	Repeated assessment centre visit								
Recruitment assessment centre visit	Prefer not to answer	Do not know	0	Less than 1	1	2	3	4	≥5
Heaped tablespoons of cooked vegetables									
Prefer not to answer	0	0	0	2	1	1	0	1	0
Do not know	2	10	9	6	19	38	18	5	5
0	0	8	149	42	86	53	29	11	18
Less than 1	0	4	55	70	124	71	23	4	7
1	1	11	97	139	1136	1070	262	79	55
2	0	19	57	74	1068	3704	1612	426	271
3	0	10	19	12	270	1720	2156	754	441
4	0	6	8	4	66	433	741	478	362
≥5	0	4	16	5	62	251	488	379	698
Heaped tablespoons of salad or raw vegetables									
Prefer not to answer	0	0	1	1	2	0	0	1	0
Do not know	1	17	24	8	41	17	12	3	6
0	0	5	820	213	434	144	39	19	35
Less than 1	0	7	254	268	372	111	24	10	20
1	5	29	629	495	3229	1301	408	156	158
2	0	25	212	158	1746	1596	713	270	247
3	1	8	76	53	582	858	629	283	270
4	0	7	28	29	240	346	336	188	211
≥5	1	12	35	25	217	332	359	283	640
Pieces of fresh fruit									
Prefer not to answer	0	0	0	0	0	1	0	0	0
Do not know	1	3	5	4	9	8	2	1	3
0	1	4	416	105	293	67	28	9	7
Less than 1	0	2	127	172	256	57	28	3	6
1	2	10	344	347	2823	1240	345	98	73
2	1	10	83	67	1561	2661	1065	265	113
3	0	2	32	30	446	1479	1515	506	279
4	0	1	11	8	113	337	587	439	285
≥5	1	1	12	6	56	154	335	318	656
Pieces of dried fruit									
Prefer not to answer	0	1	3	0	0	0	0	0	0
Do not know	1	16	41	17	30	12	5	0	6
0	2	32	6985	747	1320	310	121	56	121
Less than 1	1	15	763	419	430	113	31	18	34
1	1	17	1296	419	2083	562	202	85	112
2	2	5	313	109	587	418	193	56	86
3	0	5	135	36	227	186	147	55	82
4	0	3	74	20	95	78	58	42	46
≥5	0	4	118	33	153	123	91	81	247
Partial fibre score in fifths†									
	Missing‡		1	2	3	4	5		
Missing‡	87		165	152	142	153	157		
1	55		1863	670	267	147	82		
2	47		1063	1153	797	413	194		
3	52		520	1053	1148	802	467		
4	52		272	665	1085	1324	919		
5	65		117	312	550	1159	2179		

* Shaded cells depict participants categorised into the same (dark shading) or adjacent (light shading) category at recruitment and at the repeat assessment visit.

† Generated from UK Biobank touchscreen question on fruit, vegetables, bread and breakfast cereals.

‡ Participants who answered ‘prefer not to answer’ of ‘do not know’ to one or more of the components of the fibre score (fruit, vegetables, bread, or breakfast cereals) were assigned to missing.

**Table 4.** Reported consumption of fish and meat among 20 348 participants who answered the dietary touchscreen questionnaire about 4 years apart*

	Repeated assessment centre visit							
Recruitment assessment centre visit	Prefer not to answer	Do not know	Never	Less than once per week	Once per week	2–4 times per week	5–6 times per week	Once or more daily
Oily fish								
Prefer not to answer	0	0	1	2	0	0	0	0
Do not know	0	2	9	21	9	1	3	0
Never	1	5	1368	345	80	34	1	0
Less than once per week	0	9	306	3902	1918	326	14	5
Once per week	0	9	71	1792	4669	1364	32	7
2–4 times per week	2	1	12	272	1415	2048	87	13
5–6 times per week	0	0	1	10	21	74	29	8
Once or more daily	1	0	0	0	5	16	7	7
Other types of fish								
Prefer not to answer	0	0	0	1	1	0	0	0
Do not know	0	4	4	17	13	0	0	0
Never	0	1	662	150	51	11	1	0
Less than once per week	0	14	154	3345	2050	255	13	5
Once per week	2	11	56	2361	6394	1367	16	3
2–4 times per week	0	2	13	333	1597	1279	36	5
5–6 times per week	0	0	0	9	24	40	7	4
Once or more daily	0	0	2	3	4	6	9	0
Processed meat								
Prefer not to answer	1	0	0	0	1	1	0	0
Do not know	0	1	2	9	3	4	0	0
Never	0	0	1490	392	91	40	3	4
Less than once per week	1	6	395	3569	1535	610	54	12
Once per week	0	4	105	1661	2477	1495	85	20
2–4 times per week	1	0	50	683	1551	2864	273	45
5–6 times per week	0	1	6	60	91	291	157	34
Once or more daily	0	0	3	10	19	52	36	36
Chicken, turkey or other poultry								
Prefer not to answer	0	0	0	0	0	1	0	0
Do not know	0	4	0	8	3	4	0	0
Never	0	1	1153	70	22	24	0	0
Less than once per week	0	3	75	1066	831	267	9	2
Once per week	1	4	28	792	4095	2419	28	3
2–4 times per week	0	3	15	232	2080	6471	243	16
5–6 times per week	0	0	3	5	25	199	84	13
Once or more daily	1	0	1	0	4	14	9	6
Beef								
Prefer not to answer	0	0	0	0	0	0	0	
Do not know	0	5	2	22	4	0	0	0
Never	0	6	1826	403	49	9	1	0
Less than once per week	0	13	409	6412	1857	313	0	1
Once per week	1	3	56	2161	3410	945	8	4
2–4 times per week	0	1	16	334	995	1020	11	1
5–6 times per week	0	0	0	5	5	18	5	0
Once or more daily	1	0	1	2	1	0	0	0
Lamb/mutton								
Prefer not to answer	0	0	0	0	0	0	0	0
Do not know	0	11	9	37	7	1	0	0
Never	1	4	2626	596	38	3	0	0
Less than once per week	2	32	763	9295	1450	82	1	1
Once per week	0	7	69	2298	2200	217	0	1
2–4 times per week	0	0	2	155	84	134	0	0
5–6 times per week	0	0	0	1	1	3	1	0
Once or more daily	1	0	0	1	1	0	0	0
Pork								
Prefer not to answer	0	0	0	3	0	0	0	0
Do not know	0	7	7	38	5	2	0	0
Never	3	4	2498	678	84	16	1	0
Less than once per week	1	25	730	8917	1876	164	4	2
Once per week	0	3	71	1958	2234	349	5	1
2–4 times per week	0	0	12	154	263	192	5	1
5–6 times per week	0	0	0	8	0	8	0	0
Once or more daily	0	0	2	0	1	1	0	1

* Shaded cells depict participants categorised into the same (dark shading) or adjacent (light shading) category at recruitment and at the repeat assessment visit.

**Table 5.** Agreement of responses to dietary touchscreen questions at the baseline and repeat assessment centre visit (κ Coefficients with quadratic weighting and 95 % confidence intervals)

			Participants who completed a repeat visit*		Participants who did not report changing their diet at repeat visit†	
Question	Percentage in same or adjacent category	Percentage in same category	κ	CI	κ	CI
Cooked vegetables	81·7	41·6	0·53	0·52, 0·55	0·56	0·54, 0·57
Raw/salad vegetables	71·8	36·7	0·53	0·52, 0·55	0·56	0·55, 0·58
Fresh fruit	81·7	42·9	0·64	0·63, 0·65	0·68	0·67, 0·69
Dried fruit	71·9	51·4	0·52	0·51, 0·54	0·56	0·54, 0·57
Fibre	79·2	39·9	0·60	0·59, 0·61	0·63	0·62, 0·64
Oily fish	95·5	59·3	0·66	0·65, 0·67	0·68	0·67, 0·69
Other fish	96·0	57·7	0·53	0·52, 0·54	0·56	0·54, 0·57
Processed meat	89·9	52·2	0·62	0·61, 0·63	0·66	0·65, 0·67
Poultry	96·6	63·4	0·71	0·70, 0·72	0·74	0·73, 0·75
Beef	96·0	62·5	0·65	0·64, 0·66	0·67	0·66, 0·69
Lamb	98·2	70·5	0·63	0·62, 0·64	0·65	0·64, 0·67
Pork	97·4	68·4	0·60	0·59, 0·61	0·63	0·61, 0·64

* For each item, people who answered ‘do not know’ or ‘prefer not to answer’ at either baseline or repeat visit were excluded. The sample size for each question is as follows: cooked vegetables, *n* 20 155; raw/salad vegetables, *n* 20 101; fresh fruit, *n* 20 263; dried fruit, *n* 20 116; fibre, *n* 19 221; oily fish, *n* 20 259; other fish, *n* 20 265; processed meat, *n* 20 300; poultry, *n* 20 302; beef, *n* 20 277; lamb, *n* 20 223; pork, *n* 20 237.

† People who, at the repeat visit, reported that they made a major change to their diet in the past 5 years, or who responded with ‘prefer not to answer’ to this question were excluded. For each item, people who answered ‘do not know’ or ‘prefer not to answer’ at either baseline or repeat visit were also excluded. The sample size for each question is as follows: cooked vegetables, *n* 13 213; raw/salad vegetables, *n* 13 181; fresh fruit, *n* 13 282; dried fruit, *n* 13 191; fibre, *n* 12 603; oily fish, *n* 13 280; other fish, *n* 13 280; processed meat, *n* 13 306; poultry, *n* 13 306; beef, *n* 13 291; lamb, *n* 13 264; pork, *n* 13 278.

### Agreement between the intakes of food groups and partial fibre score estimated from the touchscreen dietary questions and group mean intakes from the 24-h dietary assessment

After averaging the values from all 24-h dietary assessments from participants who completed more than one 24-h dietary assessment, the mean daily intakes were 217 g for vegetables, 202 g for fresh fruit, 16·3 g for fibre, 11 g for oily fish, 16 g for white fish, 31 g for poultry, 58 g for red and processed meat, and 92 g for total meat. For women the mean intakes were 237 g for vegetables, 213 g for fresh fruit, 16·1 g for fibre, 12 g for oily fish, 15 g for other fish, 31 g for poultry, 50 g for red and processed meat, and 84 g for total meat. For men they were 191 g for vegetables, 189 g for fresh fruit, 16·6 g for fibre, 11 g for oily fish, 16 g for white fish, 31 g for poultry, 67 g for red and processed meat, and 102 g for total meat. For all foods and food groups, the comparisons with the 24-h dietary assessments that were completed at the assessment centre showed good agreement, with slight regression to the mean (i.e. a narrower range of intakes from the low to high categories). The comparison with the online 24-h dietary assessments showed greater regression to the mean (Table 6 and Supplementary Table S5).

**Table 6.** Comparison of the touchscreen estimate of food group intakes and partial fibre score with the group mean intakes from the 24-h dietary assessments

Intake at recruitment (touchscreen questionnaire)	Mean intake (g/d) from the recruitment 24-h dietary assessment only (*n* 70 046)	Mean intake (g/d) from the online 24-h dietary assessments (*n* 175 402)*
Total vegetables (servings per d) (*n* 491 682)		
Less than 2	148	164
2·00–2·99	223	219
3·00–3·99	272	254
4 or more	326	293
Fruit (pieces per d) (*n* 499 398)		
Less than 1	25	61
1·00–1·99	103	118
2·00–2·99	186	180
3 or more	316	274
Dried fruit (pieces per d) (*n* 495 678)		
Less than 1	3	6
1·00–1·99	17	14
2·00–2·99	28	20
3 or more	45	28
Total fruit† (servings per d) (*n* 494 578)		
Less than 2	88	109
2·00–2·99	190	183
3·00–3·99	259	234
4 or more	365	314
Total fruit and vegetables (servings per d) (*n* 490 892)		
Less than 3	206	243
3·00–3·99	321	328
4·00–4·99	426	412
6 or more	603	547
Partial fibre score, fifths, mean (sd) (*n* 478 901)		
Lowest fifth, 6·8 (sd 1·9) g/d	12·0	12·9
2, 10·9 (sd 0·9) g/d	14·8	14·9
3, 13·7 sd (0·8) g/d	16·5	16·2
4, 16·8 (sd 1·0) g/d	18·1	17·4
Highest fifth, 23·5 (sd 6·1) g/d	21·0	19·6
Oily fish (frequency per week) (*n* 498 558)		
Never	0·4	1·2
Less than once	4·7	7·2
Once	11·5	12·6
2 or more times	27·1	22·5
Other fish (frequency per week) (*n* 498 914)		
Never	1·4	2·1
Less than once	9·7	11·7
Once	17·1	16·4
2 or more times	29·2	23·0
Processed meat (frequency per week) (*n* 500 408)		
Never	2·8	4·9
Less than once	12·7	16·2
Once	19·7	21·7
2 or more times	30·1	29·2
Poultry (frequency per week) (*n* 500 565)		
Never	0·3	1·6
Less than once	14·0	19·1
Once	24·1	28·3
2 or more times	41·5	39·5
Beef (frequency per week) (*n* 499 314)		
Never	2·2	4·1
Less than once	18·1	20·9
Once	29·6	28·5
2 or more times	40·4	35·7
Lamb (frequency per week) (*n* 498 152)		
Never	0·4	1·0
Less than once	5·8	6·0
Once	13·8	10·9
2 or more times	24·8	18·6
Pork (frequency per week) (*n* 498 342)		
Never	0·4	1·5
Less than once	6·8	8·0
Once	14·8	13·7
2 or more times	23·4	19·0
Total red meat‡ (frequency per week) (*n* 495 637)		
Less than once	5·6	7·7
1·00–1·99	30·1	33·7
2·00–2·99	43·7	43·7
3 or more times	57·9	53·9
Total red and processed meat (frequency per week) (*n* 495 023)		
Less than twice	16·5	21·3
2·00–2·99	47·1	52·0
3·00–3·99	61·6	64·1
4 or more times	75·6	75·5
Total meat§ (frequency per week) (*n* 494 644)		
Less than 4 times	53·2	60·5
4·00–5·99	91·0	96·0
6·00–7·99	105·0	107·9
8 or more times	120·7	121·0

* Group mean intakes from the 24-h dietary assessments completed online.

† One piece of fresh fruit is equal to one serving of fruit, and two pieces of dried fruit are equal to one serving of fruit.

‡ Total red meat is the sum of beef, lamb and pork from the touchscreen questions.

§ Total meat is the sum of processed meat, poultry, beef, lamb and pork from the touchscreen questions.

## Discussion

In the present paper, we describe the reproducibility of the touchscreen questionnaire in the subsample of approximately 20 000 participants who completed it about 4 years apart. For the main food groups, fruit, vegetables, fish, meat, as well as the new partial fibre score, there was moderate to substantial agreement between the responses to the dietary touchscreen questions at baseline and the repeat visit. We also compared the touchscreen questionnaire, which typically asked about the frequency of consumption of the main food groups, with the data from the 24-h dietary assessment, which gives an estimate of actual intakes of foods and nutrients and was completed at least once by about 210 000 participants, by categorising participants based on the answers to the touchscreen questionnaire and calculating the group mean intake from the 24-h dietary assessments within each category. For all foods and food groups, as well as for the partial fibre score, the comparison with the group mean intakes from the 24-h dietary assessment data showed that the touchscreen questionnaire categorisation reliably ranked participants by the estimated average intake.

We have confirmed for the major food groups that participants stay relatively stable in terms of the assessment of dietary exposures during 4 years of follow-up. The questions on the consumption of meat and oily fish from the touchscreen questionnaire, particularly, showed substantial agreement with the repeat measure approximately 4 years later. The questions on the consumption of fresh fruit showed substantial agreement while the question for dried fruit showed moderate agreement. In our cohort, a high proportion of participants reported consuming dried fruit less than once daily (61 %), whereas only a small proportion (9 %) reported consuming fresh fruit less than daily; foods eaten infrequently tend to have relatively lower reproducibility than foods eaten more frequently^(^^11^^,^^12^^)^. We found similar reproducibility of the dietary touchscreen questionnaire by sex and age. However, we observed systematically better agreement in the dietary touchscreen questionnaires for participants who had a BMI < 25 kg/m^2^, compared with those with a BMI > 25 kg/m^2^. Other studies have found some differences in the reproducibility of an FFQ between normal-weight and overweight participants for foods^(^^12^^)^ and nutrients^(^^13^^)^, but the differences were inconsistent and the reproducibility was not systematically worse among overweight participants. The poorer reproducibility of the dietary touchscreen questions among overweight participants should be considered in future UK Biobank studies. A 4-year period between administrations of the touchscreen questionnaire allows us to examine the long-term reproducibility of the questionnaire – any changes will be due to a combination of variability in response to the questions and true dietary changes over time, both of which contribute to misclassification of long-term dietary intakes^(^^3^^)^. Examining this longer-term reproducibility is vital for future prospective work from UK Biobank on diet and disease development and we have shown that over 4 years of follow-up that the vast majority (>70 %) of participants report the same or adjacent category of consumption for the main food groups of fruit, vegetables, meat and fish, as well as our derived estimated partial fibre score (in fifths).

The mean daily intakes of the main food groups from all 24-h dietary assessments in UK Biobank were similar to or slightly higher than those of the same food groups from the UK National Diet and Nutrition Survey (NDNS) for adults aged 19 years or older, which is not unexpected given the known under-reporting of energy intakes in NDNS, by a magnitude of approximately 30 %^(^^14^^)^, and the non-representativeness of the UK Biobank cohort^(^^15^^)^. In addition, the age categories in NDNS (19–64 years, and 65 years or older) are wider than the age range of participants in UK Biobank (40–69 years). The mean intake of vegetables from the 24-h dietary assessments in UK Biobank was 217 g compared with 183–186 g for adults aged 19 years or older in NDNS, for fresh fruit it was 202 g in UK Biobank and 96–127 g for fresh/canned fruit in NDNS, for oily fish it was 11 g compared with 8–12 g, for white fish it was 16 g compared with 12–16 g, for poultry it was 31 g compared with 23–38 g, for red and processed meat it was 58 g compared with 63–71 g, and for total meat it was 92 g compared with 86–109 g^(^^14^^)^.

The comparison of the touchscreen dietary variables with the group mean intakes from the 24-h dietary assessments showed that the touchscreen dietary questions discriminate between low and high intakes of main food groups. The comparison also showed classic regression to the mean. This occurs because participants will randomly over- and under- report on the touchscreen questionnaire; when participants are categorised based on the answers to the touchscreen questions the lowest category will include a disproportionate number of people who reported an intake lower than their true intake, and the top category will include a disproportionate number of people who reported an intake higher than their true intake, thus the mean intakes from the 24-h dietary assessment re-measurement for each category will be closer together^(^^16^^)^. For the comparison with the group mean intakes from the 24-h dietary assessment completed at the recruitment centre, as expected, there is less regression to the mean because the two measures were completed on the same day. The new variables that we generated, of food groups in weight amounts from the 24-h dietary assessments, can be used to correct for regression dilution bias in diet–disease analyses. This can be done using the approach we have shown in this paper, by grouping participants according to baseline intakes reported at the touchscreen and calculating the mean intakes from the 24-h dietary assessments within each group. The relative risk of disease can be reported for each category of intake, and the data from the 24-h dietary assessments can be used to generate the trends in risk per increment (in g/d) in dietary intake.

The touchscreen questionnaire included questions on fruit, vegetables, bread and breakfast cereals, and from this we were able to estimate a partial fibre score for the whole cohort. According to the NDNS, the food groups in the touchscreen questionnaire that were used to estimate the fibre score contribute 54–60 % of the total fibre intakes for this age group. The other categories in NDNS that were major sources of fibre but were not asked about in the touchscreen were: potatoes; pasta, rice, pizza and other miscellaneous cereals; and biscuits; buns, cakes, pastries and fruit pies; which together contributed another 22–25 % to fibre intakes for adults aged 19 years and over^(^^14^^)^. Therefore, our estimated partial fibre score from the touchscreen is not a complete estimate of fibre intake. For epidemiological studies that investigate the associations between dietary intakes and health outcomes it is not necessary to determine food and nutrient intakes with absolute accuracy, but it is important to demonstrate that the questionnaire can discriminate between people with low and high intakes; the comparison of the touchscreen partial fibre score with the group mean intakes from the 24-h dietary assessments confirmed that the touchscreen partial fibre score that we derived does separate UK Biobank participants with low and high intakes of dietary fibre. This variable will be returned to UK Biobank, and there will now be an estimated partial fibre score for the whole cohort, which can be used to assess the relationships between fibre and disease. The partial fibre score should not be regarded as a measure of absolute fibre intake and therefore it should not be used for direct comparison with recommended intakes or intakes in other populations.

This work has shown that the main dietary touchscreen variables, including the new partial fibre score, show moderate to substantial reproducibility over a 4-year period, and comparison with the mean intakes from the 24-h dietary assessments showed that the touchscreen variables reliably rank participants according to the intake of main foods and food groups. This work underlies future research examining diet–disease associations in UK Biobank.

## Supplementary material

For supplementary material accompanying this paper visit https://doi.org/10.1017/jns.2017.66. click here to view supplementary material
