# Supplementary material for: Dietary assessment in UK Biobank: an evaluation of the performance of the touchscreen dietary questionnaire
Source: J Nutr Sci. 2018 Feb 1;7:e6. doi: 10.1017/jns.2017.66 (PMC5799609; doi:10.1017/jns.2017.66)
Supplement: Supplementary file 1 [file S2048679017000660sup001.doc]

**Supplementary materials**

**Contents:**

**Supplementary Methods page 2**

**Description of the dietary touchscreen questionnaires**

**and possible responses page 2**

**Generation of the partial fibre score from the touchscreen page 3**

**Supplementary Table S1.** Fibre content of the dietary touchscreen

questions used to estimate a dietary fibre score in UK Biobank **page 5**

**Supplementary Table S2.** Dietary variables from the Oxford WebQ

24-hour dietary assessment included in each touchscreen food group **page 7**

**Supplementary Table S3.** Agreement of responses to dietary

touchscreen questions at the baseline and repeat assessment centre

visit by sex, age, and BMI **page 9**

**Supplementary Table S4.** Reported consumption of other dietary

variables among 20,348 participants who answered the dietary

touchscreen questionnaire about 4 years apart **page 11**

**Supplementary Table S5.** Comparison of the touchscreen estimate

of food group intakes with the mean group intakes from the 24-hour

dietary assessments **page 20**

**Supplementary References page 25**

**Supplementary Methods**

**Description of the dietary touchscreen questionnaires and possible responses**

For vegetables and fruit, participants were asked to direct enter the number of heaped tablespoons (cooked vegetables, salad/raw vegetables) or pieces (fresh fruit, dried fruit, with examples given as to what constitutes a piece) eaten per day or select ‘less than one’, ‘do not know’ or ‘prefer not to answer’. For oily fish, other fish, processed meats, poultry, beef, lamb, pork and cheese, no portion size was given in the question, participants were asked how often each item was consumed with possible answers being: ‘never, ‘less than once a week’, ‘2-4 times a week’, ‘5-6 times a week’, ‘once or more daily’, ‘do not know’, ‘prefer not to answer’. For salt added to food, participants were asked if they added salt to their food, with possible answers being ‘never/rarely’, ‘sometimes’, ‘usually’, ‘always’, ‘prefer not to answer’. For amount of tea, coffee and water, participants were asked to direct enter how many cups of tea, including black and green tea, coffee (including decaffeinated coffee) or glasses of water they drank each day, or select ‘less than one’, ‘do not know’, or ‘prefer not to answer’. For type of coffee participants were asked to select the type they usually drink from ‘decaffeinated coffee (any type)’, ‘instant coffee’, ‘ground coffee (include espresso, filter etc), ‘other type of coffee’, ‘do not know’, ‘prefer not to answer’. For type of milk participants were asked to select the type of milk they mainly used out of ‘full cream’, ‘semi-skimmed’, ‘skimmed’, ‘soya’, ‘other type of milk’, ‘never/rarely have milk’, ‘do not know’, ‘prefer not to answer’. For type of spread, participants were asked firstly to select the type of spread they mainly used out of ‘butter/spreadable butter’, ‘other type of spread/margarine’, ‘never/rarely use spread’, ‘do not know’ or ‘prefer not to answer’, for those that chose ‘other type of spread/margarine’ or ‘do not know’ they were then asked a second question about the type of spread mainly used, with possible answers being ‘soft (tub) margarine’, ‘hard (block) margarine’, ‘olive oil based spread (eg: Bertolli)’, ‘Polyunsaturated/sunflower oil based spread (eg: Flora)’, ‘Flora Pro-Active or Benecol’, ‘other low or reduced fat spread’, ‘other type of spread/margarine’, ‘do not know’, ‘prefer not to answer’. For the questions on the amount of bread and amount of cereal participants were asked to direct enter the number of slices of bread eaten and the number of bowls of cereal eaten per week, or select select ‘less than one’, ‘do not know’, or ‘prefer not to answer’. For bread type, participants were asked what type they mainly ate, with possible answers being ‘white’, ‘brown’, ‘wholemeal or wholegrain’, ‘other type of bread’, ‘do not know’ or ‘prefer not to answer’. For cereal type, participants were asked what type they mainly ate, with possible answers being ‘bran cereal (e.g. All Bran, Branflakes)’, ‘biscuit cereal (e.g. Weetabix)’, oat cereal (e.g. Ready Brek, porridge)’ ‘muesli’, ‘other (e.g. Cornflakes, Frosties), ‘do not know’, ‘prefer not to answer’. For avoidance of foods, participants were asked which of the following foods they never ate, with possible answers being ‘eggs or foods containing eggs’, ‘dairy products’, ‘wheat products’, ‘sugar or foods/drinks containing sugar’, ‘I eat all of the above’, ‘prefer not to answer’. For age at last ate meat, participants were asked to direct enter how old they were when they last ate any kind of meat, to enter ‘0’ if they have never eaten meat in their lifetime, or select ‘do not know’, or ‘prefer not to answer’. For hot drink temperature, participants were asked how they liked their hot drinks, with possible answers being ‘very hot’, ‘hot’, ‘warm’, ‘do not drink hot drinks’, ‘prefer not to answer’. For changes in diet, participants were asked if they made any major changes to their diet in the past 5 years with possible answers being ‘no’, ‘yes, because of illness’, ‘yes, because of other reasons’, ‘prefer not to answer’. For variation in diet, participants were asked if their diet varied much from week to week, with possible answers being ‘never/rarely’, ‘sometimes’, ‘often’, ‘do not know’, ‘prefer not to answer’.

**Generation of the partial fibre score from the touchscreen**

We assigned pieces of fruit, tablespoons of vegetables, slices of bread, and bowls of breakfast cereals a portion size in grams based on standard portion sizes^(6)^, and an approximate non-starch polysaccharide content, based on McCance and Widdowson’s The Composition of Foods (Seventh Summary Edition)^(7)^. To calculate the fibre score we multiplied the fibre content by the frequency of consumption for each food (‘less than one’ was coded as 0.5). For bread and breakfast cereal for which the touchscreen questionnaire asked about weekly consumption, the results were converted into a daily average. We then summed the fibre intakes to get a daily estimated partial fibre intake. Participants who selected ‘do not know’ or ‘prefer not to answer’ for bread type or breakfast cereal type, but who reported their frequency of consumption of these food items, were assigned the average fibre content of these items. For fresh fruit, dried fruit, raw vegetables, cooked vegetables, bread frequency and breakfast cereal frequency, if participants selected ‘do not know’ or ‘prefer not to answer’ for any of the questions used in the fibre calculation, they were coded as missing for the estimated partial fibre score (n = 49,079). **Supplementary Table S1** shows the estimated fibre content of the touchscreen questions used to generate the partial fibre score. The mean (SD) daily intake for the estimated partial fibre score from the touchscreen was 14.3 g (6.4 g) in all participants, and 14.6 g (6.1 g) in women and 14.0 g (6.7 g) in men.

| **Supplementary Table S1.** Fibre content of the dietary touchscreen questions used to estimate a partial dietary fibre score in UK Biobank | | | | | | | |
| --- | --- | --- | --- | --- | --- | --- | --- |
| Food item (UK Biobank variable number) | Portion specified in touchscreen question | | Portion size (7) | Estimated non-starch polysaccharide content g/100g (8) | Estimated fibre content/portion (g) | Comments | |
| Fresh fruit (n_1309) | Pieces (1 apple, 1 banana, 10 grapes, etc) | | Apple: 100 g; banana: 100 g; orange: 160 g; pear: 170 g | Apple: 1.3; banana: 0.8; orange: 1.7; pear: 1.6 | 2.0 | Food codes: for apple, 14-319; for banana, 14-318; for orange, 14-327; for pear, 14-321. Approximately 2 g per piece of fruit, on average | |
| Dried fruit (n_1319) | Pieces (1 apricot, 1 prune, 10 raisins) | | Apricot: 8 g; Prune: 10g; Raisins: 10 raisins = 15 g* | Prunes: 5.7 g; Raisins: 2.0 g; Apricots: 7.7 g (Fruit and Nuts the first supplement 5th ed) | 0.5 | Food codes: 14-031 for dried apricots (from the first supplement to the 5th ed); for prunes, 14-239; for raisins,14-242. Approximately 0.5 g per piece of dried fruit, on average | |
| Cooked vegetables (n_1289) | Heaped tablespoons | | Baked beans: 40 g; peas: 30g; carrots: 40g sweetcorn: 30 g | Baked beans: 3.8 g; peas: 4.0 g; carrots: 2.1 g; sweetcorn: 2.6 g | 1.0 | Food codes: for baked beans, 13-532; for peas, 13-536; for carrots, 13-497; for sweetcorn, 13-508. Approximately 1 g per tablespoon, on average | |
| Raw vegetables (n_1299) | Heaped tablespoons | | † |  | 1.0 | Assigned the same approximate fibre content as cooked vegetables | |
| Bread type (n_1448) Bread intake (n_1438)‡ | Slices | | White bread: 36 g | 1.9 | 0.68 | Food code: 11-980 | |
|  |  |  | Brown bread: 36 g | 3.5 | 1.26 | Food code: 11-971 | |
|  |  |  | Wholemeal bread: 36 g | 5.0 | 1.80 | Food code: 11-981 | |
|  |  |  | Other/do not know/prefer not to answer |  | 1.25 | Average of the three bread types | |
| Breakfast cereal type (n_1468) Breakfast cereal intake (n_1458)‡ | Bowls | | Bran cereal: 40 g | 17.9 | 7.16 | Average of food codes: 11-767 and 11-763 | |
|  |  |  | Biscuit cereal: 40 g | 7.3 | 2.92 | Food code: 11-773 | |
|  |  |  | Oat cereal: 160 g | 1.2 | 1.92 | Average of food codes 11-793, 11-795, and 11-789 | |
|  |  |  | Muesli: 55 g | 7.6 | 4.18 | Average of food codes 11-780 and 11-781 | |
|  |  |  | Other (e.g. cornflakes): 30 g | 1.8 | 0.54 | Food code: 11-742 | |
|  |  |  | Do not know/prefer not to answer |  | 3.34 | Average of the five cereal types | |
| *1 Tablespoon of raisins weighs 30g and holds 20 raisins | | | | | | | |
| †Weight for raw vegetables assumed to be the same as for cooked vegetables | | | | | | | |
| ‡Participants who answered 0 or "Less than one" for slices of bread and bowls of breakfast cereal were not asked which type of bread or cereal they usually consumed so they were given 0 g of fibre from these questions | | | | | | | |
|  |  |  |  |  |  |  |  |
|  |  | |  |  |  |  | |
| **Supplementary Table S2.** Dietary variables from the Oxford WebQ 24-hour dietary assessment included in each touchscreen food group | | | | | | |  |
| Touchscreen food group (description, variable number) | | Dietary variables from the 24-hour dietary assessment included in each food group | | | | |  |
| Total vegetables: sum of cooked vegetables (n_1289) and raw/salad vegetables (n_1299) | | Baked beans, pulses, mixed vegetables, vegetables pieces, coleslaw, salad, avocado, broadbeans, green beans, beetroot, broccoli, squash, cabbage, carrots, cauliflower, celery, courgette, cucumber, garlic, leeks, lettuce, mushrooms, onion, parsnip, peas, peppers, spinach, sprouts, corn, sweet potato, fresh tomato, tinned tomato, turnip, watercress, other vegetables, the avocado disaggregated from guacamole using a standard recipe, the vegetables disaggregated from vegetable and pea/bean/lentil canned or home-made soup, the vegetables disaggregated from hummus, and the vegetables disaggregated from tomato-based pasta sauce | | | | |  |
| Fresh fruit (n_1309) | | Mixed fruit, apple, banana, berries, cherries, grapefruit, grapes, mango, melon, orange, satsuma, peach, pear, pineapple, plum, other fruit | | | | |  |
| Dried fruit (n_1319) | | Prunes, dried fruit, and dried fruit disaggregated from dried fruit in breakfast cereal (for participants who answered "yes" to "Did you cereal contain any dried fruit"?"; if participants answered "varied", half of the total amount was used) | | | | |  |
| Oily fish (n_1329) | | Oily fish | | | | |  |
| Other fish (n_1339) | | Tinned tuna, breaded fish, battered fish, white fish | | | | |  |
| Processed meat (n_1349) | | Sausage, crumbed chicken, bacon, ham | | | | |  |
| Poultry (n_1359) | | Chicken | | | | |  |
| Beef (n_1369) | | Beef | | | | |  |
| Lamb/mutton (n_1379) | | Lamb | | | | |  |
| Pork (n_1389) | | Pork | | | | |  |
| Red meat (sum of beef, lamb/mutton, pork) | | Beef, pork, lamb | | | | |  |
| Total meat (sum of processed meat, poultry, beef, lamb/mutton, pork) | | Beef, pork, lamb, sausage, crumbed chicken, bacon, ham, chicken, liver, other meat | | | | |  |
| Cheese (n_1408) | | Low fat hard cheese, hard cheese, soft cheese, blue cheese, low fat cheese spread, cheese spread, cottage cheese, feta, mozzarella, goat cheese, other cheese, and the cheese disaggregated from cheese sauce using the recipe provided in McCance and Widdowson’s The Composition of Food (8), cheese disaggregated from pizza using standard topping sizes, and cheese disaggregated from cheesecake using standard recipes. | | | | |  |
| Tea (n_1488) | | Black tea, rooibos, green tea, herbal tea, other tea | | | | |  |
| Caffeinated coffee (n_1508; sum of instant, ground, other type of coffee) | | Instant, filter/Americano/cafetiere, cappuccino, latte, espresso, other coffee drinks if participants consumed coffee and answered "no" to "Was it decaffeinated coffee?". If participants answered "varied"; half of their total coffee amount was assigned to caffeinated coffee | | | | |  |
| Decaffeinated coffee (n_1508) | | Instant, filter/Americano/cafetiere, cappuccino, latte, espresso, other coffee drinks if participants consumed coffee and answered "yes" to "Was it decaffeinated coffee?"; if participants answered "varied", half of their total coffee amount was assigned to decaffeinated coffee | | | | |  |
| Water (n_1528) | | Glasses of water | | | | |  |
| Slices of white bread (n_1438 and n_1448) | | Sliced white bread. For participants that selected more than one type of bread out of white, granary/brown/mixed flours/white with extras, and wholemeal, the total number of slices of bread were averaged between their selected bread types. For example, if someone consumed 6 slices of bread and selected white and wholemeal bread, they were assigned 3 slices of white bread and 3 slices of wholemeal bread | | | | |  |
| Slices of brown bread (n_1438 and n_1448) | | Sliced granary, brown, mixed flours/grains, white with extras | | | | |  |
| Slices of wholemeal/wholegrain bread (n_1438 and n_1448) | | Sliced wholemeal bread | | | | |  |
| Bran breakfast cereal (n_1468) | | Bran cereal | | | | |  |
| Biscuit cereal (n_1468) | | Wholewheat cereal | | | | |  |
| Oat cereal (n_1468) | | Porridge | | | | |  |
| Muesli (n_1468) | | Muesli | | | | |  |
| Other cereal (n_1468) | | Oat crunch, sweet cereal, plain cereal, other cereal | | | | |  |

| **Supplementary Table S3.** Agreement of responses to dietary touchscreen questions at the baseline and repeat assessment centre visit by sex, age, and BMI | | | | | | | | | | | |
| --- | --- | --- | --- | --- | --- | --- | --- | --- | --- | --- | --- |
|  | Kappa (CI) with quadratic weighting | | | | | | | | | | |
|  | Sex* | | |  | Age† | | |  | BMI‡ | | |
| Question | Men |  | Women |  | Age < 55 years |  | Age ≥ 55 years |  | BMI < 25kg/m2 |  | BMI ≥ 25kg/m2 |
| Cooked vegetables | 0.53 (0.51-0.55) |  | 0.54 (0.52-0.56) |  | 0.56 (0.54-0.58) |  | 0.51 (0.50-0.53) |  | 0.56 (0.55-0.58) |  | 0.52 (0.50-0.53) |
| Raw/salad vegetables | 0.52 (0.50-0.54) |  | 0.53 (0.51-0.54) |  | 0.53 (0.51-0.55) |  | 0.54 (0.53-0.55) |  | 0.57 (0.56-0.59) |  | 0.51 (0.50-0.53) |
| Fresh fruit | 0.64 (0.63-0.66) |  | 0.62 (0.60-0.63) |  | 0.63 (0.61-0.65) |  | 0.64 (0.63-0.65) |  | 0.67 (0.65-0.68) |  | 0.62 (0.61-0.63) |
| Dried fruit | 0.54 (0.52-0.56) |  | 0.50 (0.48-0.52) |  | 0.48 (0.45-0.50) |  | 0.54 (0.52-0.55) |  | 0.56 (0.54-0.58) |  | 0.49 (0.47-0.51) |
| Fibre | 0.61 (0.60-0.63) |  | 0.58 (0.56-0.59) |  | 0.60 (0.58-0.61) |  | 0.59 (0.58-0.60) |  | 0.63 (0.61-0.64) |  | 0.58 (0.56-0.59) |
| Oily fish | 0.66 (0.65-0.67) |  | 0.66 (0.64-0.67) |  | 0.64 (0.63-0.66) |  | 0.65 (0.64-0.67) |  | 0.67 (0.65-0.68) |  | 0.65 (0.64-0.66) |
| Other fish | 0.51 (0.49-0.53) |  | 0.55 (0.54-0.57) |  | 0.54 (0.52-0.56) |  | 0.52 (0.51-0.54) |  | 0.57 (0.55-0.59) |  | 0.51 (0.49-0.52) |
| Processed meat | 0.58 (0.57-0.60) |  | 0.60 (0.58-0.61) |  | 0.64 (0.62-0.66) |  | 0.61 (0.60-0.62) |  | 0.66 (0.64-0.68) |  | 0.59 (0.57-0.60) |
| Poultry | 0.67 (0.65-0.68) |  | 0.74 (0.72-0.75) |  | 0.73 (0.71-0.74) |  | 0.69 (0.68-0.70) |  | 0.75 (0.74-0.76) |  | 0.67 (0.65-0.68) |
| Beef | 0.61 (0.60-0.63) |  | 0.67 (0.66-0.69) |  | 0.66 (0.64-0.67) |  | 0.64 (0.63-0.65) |  | 0.69 (0.68-0.71) |  | 0.61 (0.60-0.62) |
| Lamb | 0.60 (0.58-0.62) |  | 0.66 (0.64-0.67) |  | 0.64 (0.62-0.66) |  | 0.62 (0.61-0.64) |  | 0.68 (0.66-0.69) |  | 0.60 (0.59-0.62) |
| Pork | 0.57 (0.55-0.58) |  | 0.63 (0.62-0.65) |  | 0.62 (0.60-0.64) |  | 0.59 (0.58-0.61) |  | 0.64 (0.62-0.66) |  | 0.58 (0.56-0.59) |
| *Excludes people who answered 'Do not know' or 'Prefer not to answer' at either baseline or repeat visit. The sample size for each question is as follows: for men, cooked vegetables, n=9,819; raw/salad vegetables, n=9,790; fresh fruit, n=9,893; dried fruit, n=9,826; fibre, n=9,387; oily fish, n=9,893; other fish, n=9,895; processed meat, n=9,915; poultry, n=9,909; beef, n=9,901; lamb, n=9,865; pork, n=9,875 and for women, cooked vegetables, n=10,336; raw/salad vegetables, n=10,311; fresh fruit, n=10,370; dried fruit, n=10,290; fibre, n=9,834; oily fish, n=10,366; other fish, n=10,370; processed meat, n=10,385; poultry, n=10,393; beef, n=10,376; lamb, n=10,358; pork, n=10,362 | | | | | | | | | | | |
| †Excludes people who answered 'Do not know' or 'Prefer not to answer' at either baseline or repeat visit. The sample size for each question is as follows: for <55 years, cooked vegetables, n=6,635; raw/salad vegetables, n=6,634; fresh fruit, n=6,676; dried fruit, n=6,639; fibre, n=6,305; oily fish, n=6,664; other fish, n=6,674; processed meat, n=6,684; poultry, n=6,686; beef, n=6,686; lamb, n=6,679; pork, n=6,664 and for ≥ 55 years, cooked vegetables, n=13,520; raw/salad vegetables, n=13,467; fresh fruit, n=13,587; dried fruit, n=13,477; fibre, n=12,916; oily fish, n=13,595; other fish, n=13,591; processed meat, n=13,616; poultry, n=13,616; beef, n=13,598; lamb, n=13,560; pork, n=13,573 | | | | | | | | | | | |
| ‡Excludes people who answered 'Do not know' or 'Prefer not to answer' at either baseline or repeat visit. The sample size for each question is as follows: for <25 kg/m^2^, cooked vegetables, n=7,469; raw/salad vegetables, n=7,445; fresh fruit, n=7,494; dried fruit, n=7,450; fibre, n=7,121; oily fish, n=7,496; other fish, n=7,497; processed meat, n=7,504; poultry, n=7,505; beef, n=7,495; lamb, n=7,486; pork, n=7,485 and for ≥ 25 kg/m^2^ years, cooked vegetables, n=12,686; raw/salad vegetables, n=12,656; fresh fruit, n=12,769; dried fruit, n=12,666; fibre, n=12,100; oily fish, n=12,763; other fish, n=12,768; processed meat, n=12,796; poultry, n=12,797; beef, n=12,782; lamb, n=12,737; pork, n=12,752 | | | | | | | | | | | |

| **Supplementary Table S4.** Reported consumption of other dietary variables among 20,348 participants who answered the dietary touchscreen questionnaire about 4 years apart | | | | | | | | | | | | | | | | | | | | | | | | | | | | | | | | | | | | | | | | | | | | | | |  |
| --- | --- | --- | --- | --- | --- | --- | --- | --- | --- | --- | --- | --- | --- | --- | --- | --- | --- | --- | --- | --- | --- | --- | --- | --- | --- | --- | --- | --- | --- | --- | --- | --- | --- | --- | --- | --- | --- | --- | --- | --- | --- | --- | --- | --- | --- | --- | --- |
|  | Repeated assessment centre visit | | | | | | | | | | | | | | | | | | | | | | | | | | | | | | | | | | | | | | | | | |  | | | |  |
| Recruitment assessment centre visit | *Age last ate meat** | | | | | | | | | | | | | | | | | | | | | | | | | | |  | | | | | | | |  | | |  | | | |  | | | |  |
|  | Prefer not to answer | Do not know | | 0-10 | | 11-20 | | | | 21-30 | | | | 31-40 | | | | | 41-50 | | | 51-60 | | ≥ 61 | | | | Percent in same or adjacent category (same category) | | | | | | | | Kappa (CI) with quadratic weighting | | | | | | |  | | | |  |
| Prefer not to answer | 0 | 1 | | 0 | | 0 | | | | 0 | | | | 0 | | | | | 0 | | | 0 | | 0 | | | | 95.5 (75.0) | | | | | | | | 0.79 (0.78-0.80) | | | | | | |  | | | |  |
| Do not know | 0 | 0 | | 0 | | 0 | | | | 3 | | | | 2 | | | | | 4 | | | 1 | | 3 | | | |  | | | |  |
| 0-10 | 0 | 2 | | 28 | | 2 | | | | 2 | | | | 3 | | | | | 2 | | | 3 | | 1 | | | |  | | | |  |
| 11-20 | 0 | 1 | | 7 | | 113 | | | | 22 | | | | 4 | | | | | 2 | | | 1 | | 0 | | | |  | | | |  |
| 21-30 | 0 | 3 | | 0 | | 13 | | | | 249 | | | | 39 | | | | | 5 | | | 4 | | 1 | | | |  | | | |  |
| 31-40 | 0 | 2 | | 2 | | 1 | | | | 38 | | | | 222 | | | | | 14 | | | 2 | | 0 | | | |  | | | |  |
| 41-50 | 0 | 1 | | 0 | | 5 | | | | 3 | | | | 38 | | | | | 109 | | | 16 | | 0 | | | |  | | | |  |
| 51-60 | 0 | 1 | | 1 | | 0 | | | | 0 | | | | 3 | | | | | 14 | | | 31 | | 2 | | | |  | | | |  |
| 61+ | 0 | 1 | | 0 | | 0 | | | | 0 | | | | 0 | | | | | 0 | | | 3 | | 5 | | | |  | | | |  |
|  |  |  | |  | |  | | | |  | | | |  | | | | |  | | |  | |  | | | |  | | | | | | | |  | | | | |  | |  | | | |  |
|  | *Dietary exclusions* | | | | |  | | | |  | | | |  | | | | |  | | |  | |  | | | |  | | | | | | | |  | | | | |  | |  | | | |  |
|  | *Dietary exclusions: egg†* | | | | |  | | | |  | | | |  | | | | |  | | |  | |  | | | |  | | | | | | | |  | | | | |  | |  | | | |  |
|  | Consumers | Excluders | | Percent in same category | |  | | | |  | | | |  | | | | |  | | |  | |  | | | |  | | | | | | | |  | | | | |  | |  | | | |  |
| Consumers | 19,594 | 205 | | 97.8 | |  | | | |  | | | |  | | | | |  | | |  | |  | | | |  | | | | | | | |  | | | | |  | |  | | | |  |
| Excluders | 244 | 236 | |  | | | |  | | | |  | | | | |  | | |  | |  | | | |  | | | | | | | |  | | | | |  | |  | | | |  |
|  |  |  | |  | |  | | | |  | | | |  | | | | |  | | |  | |  | | | |  | | | | | | | |  | | | | |  | |  | | | |  |
|  | *Dietary exclusions: wheat†* | | | | |  | | | |  | | | |  | | | | |  | | |  | |  | | | |  | | | | | | | |  | | | | |  | |  | | | |  |
|  | Consumers | Excluders | | Percent in same category | |  | | | |  | | | |  | | | | |  | | |  | |  | | | |  | | | | | | | |  | | | | |  | |  | | | |  |
| Consumers | 19,595 | 292 | | 97.6 | |  | | | |  | | | |  | | | | |  | | |  | |  | | | |  | | | | | | | |  | | | | |  | |  | | | |  |
| Excluders | 188 | 204 | |  | | | |  | | | |  | | | | |  | | |  | |  | | | |  | | | | | | | |  | | | | |  | |  | | | |  |
|  |  |  | |  | |  | | | |  | | | |  | | | | |  | | |  | |  | | | |  | | | | | | | |  | | | | |  | |  | | | |  |
|  | *Dietary exclusions: dairy†* | | | | |  | | | |  | | | |  | | | | |  | | |  | |  | | | |  | | | | | | | |  | | | | |  | |  | | | |  |
|  | Consumers | Excluders | | Percent in same category | |  | | | |  | | | |  | | | | |  | | |  | |  | | | |  | | | | | | | |  | | | | |  | |  | | | |  |
| Consumers | 19,675 | 240 | | 97.9 | |  | | | |  | | | |  | | | | |  | | |  | |  | | | |  | | | | | | | |  | | | | |  | |  | | | |  |
| Excluders | 186 | 178 | |  | | | |  | | | |  | | | | |  | | |  | |  | | | |  | | | | | | | |  | | | | |  | |  | | | |  |
|  |  |  | |  | |  | | | |  | | | |  | | | | |  | | |  | |  | | | |  | | | | | | | |  | | | | |  | |  | | | |  |
|  | *Dietary exclusions: sugar†* | | | | |  | | | |  | | | |  | | | | |  | | |  | |  | | | |  | | | | | | | |  | | | | |  | |  | | | |  |
|  | Consumers | Excluders | | Percent in same category | |  | | | |  | | | |  | | | | |  | | |  | |  | | | |  | | | | | | | |  | | | | |  | |  | | | |  |
| Consumers | 14,897 | 1,860 | | 84.0 | |  | | | |  | | | |  | | | | |  | | |  | |  | | | |  | | | | | | | |  | | | | |  | |  | | | |  |
| Excluders | 1,381 | 2,141 | |  | | | |  | | | |  | | | | |  | | |  | |  | | | |  | | | | | | | |  | | | | |  | |  | | | |  |
|  |  |  | |  | |  | | | |  | | | |  | | | | |  | | |  | |  | | | |  | | | | | | | |  | | | | |  | |  | | | |  |
|  | *Cheese* | | | | | | | | | | | | | | | | | | | | | | |  | | | |  | | | | | | | |  | | | | |  | |  | | | |  |
|  | Prefer not to answer | Do not know | | Never | | Less than once a week | | | | Once a week | | | | 2-4 times a week | | | | 5-6 times a week | | | | Once or more daily | | Percent in same or adjacent category (same category) | | | | Kappa (CI) with quadratic weighting | | | | | | | |  | | | | |  | |  | | | |  |
| Prefer not to answer | 0 | 0 | | 0 | | 0 | | | | 0 | | | | 0 | | | | 0 | | | | 0 | | 89.5 (52.5) | | | | 0.62 (0.61-0.63) | | | | | | | |  | | | | |  | |  | | | |  |
| Do not know | 0 | 1 | | 0 | | 6 | | | | 3 | | | | 6 | | | | 1 | | | | 3 | |  | | | | |  | |  | | | |  |
| Never | 0 | 1 | | 286 | | 90 | | | | 18 | | | | 22 | | | | 6 | | | | 1 | |  | | | | |  | |  | | | |  |
| Less than once a week | 0 | 8 | | 94 | | 1,485 | | | | 843 | | | | 608 | | | | 41 | | | | 20 | |  | | | | |  | |  | | | |  |
| Once a week | 0 | 2 | | 27 | | 895 | | | | 1,562 | | | | 1,491 | | | | 81 | | | | 18 | |  | | | | |  | |  | | | |  |
| 2-4 times a week | 2 | 2 | | 16 | | 656 | | | | 1,607 | | | | 5,891 | | | | 925 | | | | 192 | |  | | | | |  | |  | | | |  |
| 5-6 times a week | 0 | 0 | | 5 | | 59 | | | | 85 | | | | 873 | | | | 778 | | | | 239 | |  | | | | |  | |  | | | |  |
| Once or more daily | 0 | 0 | | 2 | | 13 | | | | 35 | | | | 164 | | | | 227 | | | | 332 | |  | | | | |  | |  | | | |  |
|  |  |  | |  | |  | | | |  | | | |  | | | |  | | | |  | |  | | | |  | | | | | | | |  | | | | |  | |  | | | |  |
|  | *Milk type* | | | | | | | | | | | | | | | | | | | | | | |  | | | |  | | | | | | | |  | | | | |  | |  | | | |  |
|  | Prefer not to answer | Do not know | | Full cream | | Semi-skimmed | | | | | | Skimmed | | | Soya | | | | Other type of milk | | | Never/ Rarely have milk | | Percent in same category | | | |  | | | | | | | |  | | | | |  | |  | | | |  |
| Prefer not to answer | 0 | 0 | | 0 | | 0 | | | | | | 0 | | | 1 | | | | 0 | | | 0 | | 82.2 | | | |  | | | | | | | |  | | | | |  | |  | | | |  |
| Do not know | 0 | 0 | | 0 | | 1 | | | | | | 0 | | | 0 | | | | 0 | | | 1 | |  | | | | | | | |  | | | | |  | |  | | | |  |
| Full cream | 0 | 0 | | 803 | | 401 | | | | | | 29 | | | 12 | | | | 17 | | | 27 | |  | | | | | | | |  | | | | |  | |  | | | |  |
| Semi-skimmed | 2 | 0 | | 238 | | 11,012 | | | | | | 902 | | | 136 | | | | 108 | | | 110 | |  | | | | | | | |  | | | | |  | |  | | | |  |
| Skimmed | 1 | 0 | | 27 | | 727 | | | | | | 3,927 | | | 67 | | | | 44 | | | 55 | |  | | | | | | | |  | | | | |  | |  | | | |  |
| Soya | 0 | 0 | | 16 | | 187 | | | | | | 81 | | | 481 | | | | 48 | | | 22 | |  | | | | | | | |  | | | | |  | |  | | | |  |
| Other type of milk | 0 | 1 | | 20 | | 77 | | | | | | 27 | | | 16 | | | | 92 | | | 15 | |  | | | | | | | |  | | | | |  | |  | | | |  |
| Never/Rarely have milk | 0 | 0 | | 16 | | 116 | | | | | | 42 | | | 18 | | | | 19 | | | 386 | |  | | | | | | | |  | | | | |  | |  | | | |  |
|  |  |  | |  | |  | | | | | |  | | |  | | | |  | | |  | |  | | | |  | | | | | | | |  | | | | |  | |  | | | |  |
|  | *Spread type‡* | | | | | | | | | | | | | | | | | | | | | | | | | | | | | | | | | | | | | | | |  | |  | | | |  |
|  | Prefer not to answer | Do not know | Never/rarely use spread | | Butter /spreadable butter | | | | | | Flora pro-active | | | Soft margarine | | | | | | Hard margarine | | | Olive oil based | | Polyunsaturated based | | | | | | | Other low or reduced fat | | | | | Other type | | | | Percent in same category | | | | | |  |
| Prefer not to answer | 1 | 0 | 0 | | 0 | | | | | | 0 | | | 0 | | | | | | 0 | | | 0 | | 1 | | | | | | | 0 | | | | | 0 | | | | 59.1 | | | | | |  |
| Do not know | 0 | 1 | 0 | | 0 | | | | | | 0 | | | 0 | | | | | | 0 | | | 0 | | 0 | | | | | | | 1 | | | | | 1 | | | |  |
| Never/rarely use spread | 0 | 0 | 1,403 | | 439 | | | | | | 96 | | | 33 | | | | | | 2 | | | 121 | | 123 | | | | | | | 48 | | | | | 22 | | | |  |
| Butter/spreadable butter | 1 | 0 | 365 | | 5,387 | | | | | | 296 | | | 135 | | | | | | 1 | | | 251 | | 244 | | | | | | | 116 | | | | | 48 | | | |  |
| Flora pro-active | 0 | 1 | 84 | | 258 | | | | | | 930 | | | 45 | | | | | | 0 | | | 139 | | 274 | | | | | | | 62 | | | | | 14 | | | |  |
| Soft margarine | 0 | 0 | 46 | | 184 | | | | | | 67 | | | 356 | | | | | | 3 | | | 91 | | 183 | | | | | | | 50 | | | | | 34 | | | |  |
| Hard margarine | 0 | 0 | 2 | | 4 | | | | | | 0 | | | 1 | | | | | | 2 | | | 1 | | 2 | | | | | | | 1 | | | | | 0 | | | |  |
| Olive oil based | 1 | 0 | 143 | | 487 | | | | | | 228 | | | 67 | | | | | | 0 | | | 1,623 | | 250 | | | | | | | 88 | | | | | 36 | | | |  |
| Polyunsaturated based | 2 | 0 | 149 | | 489 | | | | | | 407 | | | 228 | | | | | | 1 | | | 377 | | 1,709 | | | | | | | 252 | | | | | 55 | | | |  |
| Other low or reduced fat | 0 | 0 | 48 | | 152 | | | | | | 74 | | | 57 | | | | | | 0 | | | 91 | | 217 | | | | | | | 216 | | | | | 27 | | | |  |
| Other type | 0 | 0 | 29 | | 72 | | | | | | 22 | | | 34 | | | | | | 2 | | | 36 | | 73 | | | | | | | 44 | | | | | 117 | | | |  |
|  |  |  |  | |  | | | | | |  | | |  | | | | | |  | | |  | |  | | | | | | |  | | | | |  | | | |  | |  | | | |  |
|  | *Slices of bread per week* | | | | | | | | | | | | | | | | | | | | | | | | | | | | | | | | | | | |  | | | |  | |  | | | |  |
|  | Prefer not to answer | Do not know | | < 1 | | 1-5 | | | | | | 6-10 | | | 11-15 | | | | 16-20 | | | 21-25 | | | 26-30 | | | | | | 31+ | | | | | | Percent in same or adjacent category (same category) | | | | | | | | Kappa (CI) with quadratic weighting | | |
| Prefer not to answer | 0 | 2 | | 0 | | 0 | | | | | | 3 | | | 1 | | | | 1 | | | 0 | | | 0 | | | | | | 0 | | | | | | 78.3 (39.4) | | | | | | | | | 0.62 (0.61-0.63) | |
| Do not know | 0 | 23 | | 4 | | 19 | | | | | | 26 | | | 24 | | | | 14 | | | 2 | | | 3 | | | | | | 3 | | | | | |
| < 1 | 0 | 4 | | 202 | | 216 | | | | | | 108 | | | 31 | | | | 14 | | | 2 | | | 5 | | | | | | 2 | | | | | |
| 1-5 | 1 | 15 | | 251 | | 1,466 | | | | | | 920 | | | 230 | | | | 65 | | | 23 | | | 10 | | | | | | 6 | | | | | |
| 6-10 | 2 | 19 | | 144 | | 1,157 | | | | | | 2,844 | | | 1,128 | | | | 321 | | | 100 | | | 52 | | | | | | 9 | | | | | |
| 11-15 | 0 | 19 | | 68 | | 411 | | | | | | 1,449 | | | 1,820 | | | | 562 | | | 211 | | | 125 | | | | | | 30 | | | | | |
| 16-20 | 0 | 14 | | 28 | | 120 | | | | | | 522 | | | 821 | | | | 660 | | | 237 | | | 166 | | | | | | 34 | | | | | |
| 21-25 | 0 | 4 | | 12 | | 58 | | | | | | 158 | | | 294 | | | | 292 | | | 278 | | | 158 | | | | | | 28 | | | | | |
| 26-30 | 0 | 7 | | 14 | | 36 | | | | | | 124 | | | 192 | | | | 241 | | | 191 | | | 267 | | | | | | 107 | | | | | |
| 31+ | 0 | 8 | | 6 | | 14 | | | | | | 28 | | | 55 | | | | 79 | | | 81 | | | 168 | | | | | | 204 | | | | | |
|  |  |  | |  | |  | | | | | |  | | |  | | | |  | | |  | | |  | | | | | |  | | | | | |  | | | |  |  | | | |  | |
|  | *Bread type* | | | | | | | | | | | | | | | | | |  | | |  | | |  | | | | | |  | | | | | |  | | | |  |  | | | |  | |
|  | Prefer not to answer | Do not know | | White | | Brown | | | | | Wholemeal or wholegrain | | | | | Other type of bread | | | Percent in same category | | |  | | |  | | | | | |  | | | | | |  | | | |  |  | | | |  | |
| Prefer not to answer | 0 | 2 | | 3 | | 0 | | | | | 1 | | | | | 0 | | | 70.2 | | |  | | |  | | | | | |  | | | | | |  | | | |  |  | | | |  | |
| Do not know | 0 | 4 | | 8 | | 5 | | | | | 25 | | | | | 3 | | |  | | |  | | | | | |  | | | | | |  | | | |  |  | | | |  | |
| White | 2 | 18 | | 2,596 | | 425 | | | | | 976 | | | | | 174 | | |  | | |  | | | | | |  | | | | | |  | | | |  |  | | | |  | |
| Brown | 0 | 5 | | 297 | | 660 | | | | | 931 | | | | | 76 | | |  | | |  | | | | | |  | | | | | |  | | | |  |  | | | |  | |
| Wholemeal or wholegrain | 1 | 24 | | 792 | | 956 | | | | | 9,600 | | | | | 445 | | |  | | |  | | | | | |  | | | | | |  | | | |  |  | | | |  | |
| Other type of bread | 1 | 3 | | 100 | | 58 | | | | | 334 | | | | | 237 | | |  | | |  | | | | | |  | | | | | |  | | | |  |  | | | |  | |
|  |  |  | |  | |  | | | | |  | | | | |  | | |  | | |  | | |  | | | | | |  | | | | | |  | | | |  |  | | | |  | |
|  | *Bowls of breakfast cereal* | | | | | | | | | | | | | | | | | | | | | | | | | | | | | | | | | | | | | | |  | | | | | |  | |
|  | Prefer not to answer | Do not know | | 0 | 0.5 | | | | 1 | | | | 2 | | | | 3 | | | | 4 | | 5 | | | 6 | | | | 7 | | | | | | 8+ | | | | Percent in same or adjacent category (same category) | | | | | | Kappa (CI) with quadratic weighting | |
| Prefer not to answer | 0 | 0 | | 1 | 0 | | | | 0 | | | | 0 | | | | 0 | | | | 0 | | 0 | | | 0 | | | | 0 | | | | | | 0 | | | | 69.2 (50.5) | | | | | | 0.64 (0.63-0.65) | |
| Do not know | 0 | 4 | | 0 | 4 | | | | 1 | | | | 3 | | | | 1 | | | | 0 | | 3 | | | 1 | | | | 3 | | | | | | 1 | | | |
| 0 | 0 | 1 | | 1,130 | 165 | | | | 100 | | | | 107 | | | | 90 | | | | 59 | | 85 | | | 47 | | | | 230 | | | | | | 7 | | | |
| 0.5 | 0 | 2 | | 248 | 173 | | | | 66 | | | | 87 | | | | 61 | | | | 34 | | 62 | | | 18 | | | | 74 | | | | | | 2 | | | |
| 1 | 0 | 1 | | 126 | 67 | | | | 117 | | | | 117 | | | | 66 | | | | 32 | | 55 | | | 24 | | | | 55 | | | | | | 3 | | | |
| 2 | 1 | 3 | | 144 | 96 | | | | 109 | | | | 225 | | | | 167 | | | | 102 | | 126 | | | 43 | | | | 148 | | | | | | 7 | | | |
| 3 | 0 | 1 | | 112 | 68 | | | | 78 | | | | 177 | | | | 234 | | | | 136 | | 170 | | | 69 | | | | 137 | | | | | | 11 | | | |
| 4 | 1 | 0 | | 85 | 46 | | | | 49 | | | | 109 | | | | 156 | | | | 158 | | 170 | | | 78 | | | | 164 | | | | | | 12 | | | |
| 5 | 1 | 0 | | 172 | 61 | | | | 60 | | | | 141 | | | | 202 | | | | 249 | | 835 | | | 305 | | | | 486 | | | | | | 10 | | | |
| 6 | 0 | 0 | | 72 | 26 | | | | 32 | | | | 53 | | | | 70 | | | | 108 | | 302 | | | 488 | | | | 508 | | | | | | 21 | | | |
| 7 | 0 | 5 | | 302 | 90 | | | | 64 | | | | 108 | | | | 153 | | | | 185 | | 478 | | | 424 | | | | 6701 | | | | | | 178 | | | |
| 8+ | 0 | 0 | | 15 | 5 | | | | 0 | | | | 5 | | | | 12 | | | | 8 | | 15 | | | 18 | | | | 246 | | | | | | 187 | | | |
|  |  |  | |  |  | | | |  | | | |  | | | |  | | | |  | |  | | |  | | | |  | | | | | |  | | | |  | | | | | |  | |
|  | *Breakfast cereal type* | | | | | | | | | | | | | | | | | | | |  | |  | | |  | | | |  | | | | | |  | | | |  | | | | | |  | |
|  | Prefer not to answer | Do not know | | Bran cereal | | Biscuit cereal | | | | | | Oat cereal | | | Muesli | | | | Other | | | | Percent in same category | | |  | | | |  | | | | | |  | | | |  | | | | | |  | |
| Prefer not to answer | 1 | 0 | | 1 | | 0 | | | | | | 2 | | | 1 | | | | 3 | | | | 57.8 | | |  | | | |  | | | | | |  | | | |  | | | | | |  | |
| Do not know | 0 | 6 | | 10 | | 20 | | | | | | 16 | | | 19 | | | | 15 | | | |  | | | |  | | | | | |  | | | |  | | | | | |  | |
| Bran cereal | 1 | 12 | | 1,469 | | 239 | | | | | | 546 | | | 341 | | | | 186 | | | |  | | | |  | | | | | |  | | | |  | | | | | |  | |
| Biscuit cereal | 2 | 11 | | 266 | | 1,296 | | | | | | 670 | | | 228 | | | | 251 | | | |  | | | |  | | | | | |  | | | |  | | | | | |  | |
| Oat cereal | 2 | 10 | | 281 | | 317 | | | | | | 2,802 | | | 411 | | | | 250 | | | |  | | | |  | | | | | |  | | | |  | | | | | |  | |
| Muesli | 0 | 15 | | 298 | | 217 | | | | | | 754 | | | 2,352 | | | | 152 | | | |  | | | |  | | | | | |  | | | |  | | | | | |  | |
| Other | 3 | 9 | | 244 | | 302 | | | | | | 493 | | | 242 | | | | 1,227 | | | |  | | | |  | | | | | |  | | | |  | | | | | |  | |
|  |  |  | |  | |  | | | | | |  | | |  | | | |  | |  | |  | | |  | | | |  | | | | | |  | | | |  | | | | | |  | |
|  | *Salt added to food* | | | | | | | | | | | | | |  | | | |  | |  | |  | | |  | | | |  | | | | | |  | | | |  | | | | | |  | |
|  | Prefer not to answer | Never/rarely | | Sometimes | | Usually | | | | | | Always | | | Percent in same or adjacent category (same category) | | | | | | Kappa (CI) with quadratic weighting | | | | |  | | | |  | | | | | |  | | | |  | | | | | |  | |
| Prefer not to answer | 0 | 0 | | 0 | | 0 | | | | | | 0 | | | 97.2 (72.6) | | | | | | 0.71 (0.70-0.72) | | | | |  | | | |  | | | | | |  | | | |  | | | | | |  | |
| Never/rarely | 1 | 10,465 | | 1,656 | | 170 | | | | | | 24 | | |  | | | |  | | | | | |  | | | |  | | | | | |  | |
| Sometimes | 0 | 1,578 | | 2,987 | | 670 | | | | | | 87 | | |  | | | |  | | | | | |  | | | |  | | | | | |  | |
| Usually | 1 | 196 | | 655 | | 1,041 | | | | | | 215 | | |  | | | |  | | | | | |  | | | |  | | | | | |  | |
| Always | 0 | 35 | | 52 | | 226 | | | | | | 276 | | |  | | | |  | | | | | |  | | | |  | | | | | |  | |
|  |  |  | |  | |  | | | | | |  | | |  | | | |  | |  | |  | | |  | | | |  | | | | | |  | | | |  | | | | | |  | |
|  | *Cups of tea daily* | | | | | | | | | | | | | | | | | | | | | | | | | | | | |  | | | | | |  | | | |  | | | | | |  | |
|  | Prefer not to answer | Do not know | | 0 | | Less than one | | | | | | 1 | | | 2 | | | | 3 | | | 4 | | | 5 | | | | 6+ | | | | | Percent in same or adjacent category (same category) | | | | Kappa (CI) with quadratic weighting | | | | | |  | |  | |
| Prefer not to answer | 0 | 0 | | 0 | | 0 | | | | | | 0 | | | 0 | | | | 0 | | | 0 | | | 0 | | | | 0 | | | | | 48.9 (80.8) | | | | | | 0.83 (0.82-0.84) | | | |  | |  | |
| Do not know | 0 | 3 | | 2 | | 0 | | | | | | 1 | | | 4 | | | | 0 | | | 5 | | | 3 | | | | 5 | | | | |  | |
| 0 | 2 | 1 | | 2,278 | | 188 | | | | | | 203 | | | 116 | | | | 52 | | | 38 | | | 17 | | | | 34 | | | | |  | |
| Less than one | 0 | 2 | | 212 | | 169 | | | | | | 137 | | | 79 | | | | 28 | | | 2 | | | 9 | | | | 12 | | | | |  | |
| 1 | 0 | 1 | | 153 | | 122 | | | | | | 653 | | | 398 | | | | 114 | | | 44 | | | 29 | | | | 25 | | | | |  | |
| 2 | 0 | 0 | | 73 | | 48 | | | | | | 330 | | | 1,142 | | | | 650 | | | 244 | | | 97 | | | | 80 | | | | |  | |
| 3 | 0 | 0 | | 43 | | 21 | | | | | | 111 | | | 545 | | | | 1,127 | | | 681 | | | 273 | | | | 166 | | | | |  | |
| 4 | 2 | 0 | | 30 | | 7 | | | | | | 52 | | | 207 | | | | 649 | | | 1,049 | | | 576 | | | | 392 | | | | |  | |
| 5 | 0 | 1 | | 24 | | 6 | | | | | | 18 | | | 80 | | | | 229 | | | 573 | | | 760 | | | | 698 | | | | |  | |
| 6+ | 1 | 6 | | 41 | | 7 | | | | | | 31 | | | 101 | | | | 163 | | | 405 | | | 710 | | | | 2745 | | | | |  | |
|  |  |  | |  | |  | | | | | |  | | |  | | | |  | | |  | | |  | | | |  | | | | |  | | | | | |  | | | |  | |  | |
|  | *Cups of coffee daily* | | | | | | | | | | | | | | | | | | | | | | | | | | |  | | | | | | | | | | | |  | | | |  | |  | |
|  | Prefer not to answer | Do not know | | 0 | | Less than one | | | | | | 1 | | | 2 | | | | 3 | | | 4 | | | 5 | | | | | | 6+ | | | | Percent in same or adjacent category (same category) | | | | | Kappa (CI) with quadratic weighting | | | |  | |  | |
| Prefer not to answer | 0 | 1 | | 0 | | 0 | | | | | | 0 | | | 1 | | | | 0 | | | 0 | | | 0 | | | | | | 0 | | | | 82.2 (49.7) | | | | | 0.78 (0.77-0.79) | | | |  | |  | |
| Do not know | 0 | 2 | | 2 | | 2 | | | | | | 3 | | | 2 | | | | 0 | | | 3 | | | 1 | | | | | | 4 | | | |  | |  | |
| 0 | 0 | 0 | | 2,860 | | 417 | | | | | | 488 | | | 122 | | | | 60 | | | 36 | | | 16 | | | | | | 29 | | | |  | |  | |
| Less than one | 0 | 0 | | 388 | | 582 | | | | | | 505 | | | 156 | | | | 31 | | | 20 | | | 13 | | | | | | 10 | | | |  | |  | |
| 1 | 0 | 2 | | 306 | | 366 | | | | | | 2,245 | | | 895 | | | | 185 | | | 71 | | | 36 | | | | | | 30 | | | |  | |  | |
| 2 | 0 | 0 | | 102 | | 93 | | | | | | 755 | | | 1,884 | | | | 684 | | | 199 | | | 56 | | | | | | 50 | | | |  | |  | |
| 3 | 1 | 0 | | 48 | | 25 | | | | | | 155 | | | 685 | | | | 1003 | | | 449 | | | 106 | | | | | | 57 | | | |  | |  | |
| 4 | 0 | 0 | | 23 | | 12 | | | | | | 73 | | | 229 | | | | 445 | | | 591 | | | 271 | | | | | | 119 | | | |  | |  | |
| 5 | 0 | 1 | | 19 | | 5 | | | | | | 38 | | | 92 | | | | 122 | | | 292 | | | 314 | | | | | | 204 | | | |  | |  | |
| 6+ | 1 | 3 | | 25 | | 10 | | | | | | 33 | | | 62 | | | | 84 | | | 169 | | | 243 | | | | | | 613 | | | |  | |  | |
|  |  |  | |  | |  | | | | | |  | | |  | | | |  | | |  | | |  | | | | | |  | | | |  | | | | |  | | | |  | |  | |
|  | *Coffee type* | | | | | | | | | | | | | | | | | |  | | |  | | |  | | | | | |  | | | |  | | | | |  | | | |  | |  | |
|  | Prefer not to answer | Do not know | | Decaffeinated | | | | Instant | | | | Ground | | | Other type | | | | Percent in same category | | |  | | |  | | | | | |  | | | |  | | | | |  | | | |  | |  | |
| Prefer not to answer | 1 | 1 | | 0 | | | 0 | | | | | 3 | | | 1 | | | | 78.4 | | |  | | |  | | | | | |  | | | |  | | | | |  | | | |  | |  | |
| Do not know | 0 | 2 | | 2 | | | 14 | | | | | 5 | | | 2 | | | |  | | |  | | | | | |  | | | |  | | | | |  | | | |  | |  | |
| Decaffeinated | 1 | 2 | | 2,178 | | | 609 | | | | | 334 | | | 38 | | | |  | | |  | | | | | |  | | | |  | | | | |  | | | |  | |  | |
| Instant | 2 | 7 | | 656 | | | 6,569 | | | | | 745 | | | 80 | | | |  | | |  | | | | | |  | | | |  | | | | |  | | | |  | |  | |
| Ground | 0 | 1 | | 198 | | | 420 | | | | | 2,936 | | | 31 | | | |  | | |  | | | | | |  | | | |  | | | | |  | | | |  | |  | |
| Other type | 0 | 0 | | 20 | | | 65 | | | | | 45 | | | 53 | | | |  | | |  | | | | | |  | | | |  | | | | |  | | | |  | |  | |
|  |  |  | |  | | |  | | | | |  | | |  | | | |  | | |  | | |  | | | | | |  | | | |  | | | | |  | | | |  | |  | |
|  | *Hot drink temperature* | | | | | | | | | | | | | |  | | | |  | | |  | | |  | | | | | |  | | | |  | | | | |  | | | |  | |  | |
|  | Prefer not to answer | Do not drink hot drinks | | Very hot | | Hot | | | | | | Warm | | | Percent in same or adjacent category (same category) | | | | | | | Kappa (CI) with quadratic weighting | | | | | | | | |  | | | |  | | | | |  | | | |  | |  | |
| Prefer not to answer | 0 | 1 | | 0 | | 5 | | | | | | 0 | | | 99.9 (79.5) | | | | | | | 0.68 (0.67-0.69) | | | | | | | | |  | | | |  | | | | |  | | | |  | |  | |
| Do not drink hot drinks | 0 | 132 | | 5 | | 37 | | | | | | 18 | | |  | | | |  | | | | |  | | | |  | |  | |
| Very hot | 1 | 5 | | 2,310 | | 1,033 | | | | | | 18 | | |  | | | |  | | | | |  | | | |  | |  | |
| Hot | 3 | 24 | | 1,069 | | 11,500 | | | | | | 862 | | |  | | | |  | | | | |  | | | |  | |  | |
| Warm | 2 | 11 | | 10 | | 1,125 | | | | | | 2,164 | | |  | | | |  | | | | |  | | | |  | |  | |
|  |  |  | |  | |  | | | | | |  | | |  | | | |  | | |  | | |  | | | | | |  | | | |  | | | | |  | | | |  | |  | |
|  | *Glasses of water daily* | | | | | | | | | | | | | | | | | | | | | | | | | | |  | | | | | | | | | | | |  | | | |  | |  | |
|  | Prefer not to answer | Do not know | | 0 | | Less than one | | | | | | 1 | | | 2 | | | | 3 | | | 4 | | | 5 | | 6+ | | | | | | Percent in same or adjacent category (same category) | | | | | | | Kappa (CI) with quadratic weighting | | | |  | |  | |
| Prefer not to answer | 0 | 0 | | 0 | | 0 | | | | | | 0 | | | 1 | | | | 0 | | | 0 | | | 0 | | 0 | | | | | | 74.8 (37.3) | | | | | | | 0.70 (0.69-0.71) | | | |  | |  | |
| Do not know | 0 | 10 | | 9 | | 13 | | | | | | 17 | | | 15 | | | | 7 | | | 7 | | | 1 | | 6 | | | | | |  | |  | |
| 0 | 1 | 3 | | 932 | | 324 | | | | | | 366 | | | 93 | | | | 24 | | | 17 | | | 6 | | 10 | | | | | |  | |  | |
| Less than one | 0 | 8 | | 453 | | 659 | | | | | | 539 | | | 163 | | | | 46 | | | 18 | | | 9 | | 17 | | | | | |  | |  | |
| 1 | 0 | 9 | | 455 | | 594 | | | | | | 1,859 | | | 889 | | | | 167 | | | 54 | | | 20 | | 33 | | | | | |  | |  | |
| 2 | 1 | 9 | | 156 | | 256 | | | | | | 1,307 | | | 1,940 | | | | 590 | | | 244 | | | 58 | | 75 | | | | | |  | |  | |
| 3 | 0 | 9 | | 61 | | 67 | | | | | | 389 | | | 1,026 | | | | 751 | | | 358 | | | 106 | | 94 | | | | | |  | |  | |
| 4 | 0 | 8 | | 25 | | 27 | | | | | | 149 | | | 511 | | | | 565 | | | 468 | | | 190 | | 157 | | | | | |  | |  | |
| 5 | 0 | 3 | | 13 | | 14 | | | | | | 48 | | | 154 | | | | 237 | | | 295 | | | 214 | | 163 | | | | | |  | |  | |
| 6+ | 1 | 7 | | 12 | | 13 | | | | | | 53 | | | 154 | | | | 206 | | | 307 | | | 275 | | 715 | | | | | |  | |  | |
|  |  |  | |  | |  | | | | | |  | | |  | | | |  | | |  | | |  | |  | | | | | |  | | | | | | |  | | | |  | |  | |
|  | *Diet vary from week to week* | | | | | | | | | | | | | |  | | | |  | | |  | | |  | |  | | | | | |  | | | | | | |  | | | |  | |  | |
|  | Prefer not to answer | Do not know | | Never/rarely | | Sometimes | | | | | | Often | | | Percent in same or adjacent category (same category) | | | | | | | Kappa (CI) with quadratic weighting | | | | |  | | | | | |  | | | | | | |  | | | |  | |  | |
| Prefer not to answer | 1 | 0 | | 1 | | 1 | | | | | | 0 | | | 98.4 (63.1) | | | | | | | 0.41 (0.40-0.42) | | | | |  | | | | | |  | | | | | | |  | | | |  | |  | |
| Do not know | 0 | 5 | | 13 | | 13 | | | | | | 2 | | |  | | | | | |  | | | | | | |  | | | |  | |  | |
| Never/rarely | 1 | 5 | | 4,186 | | 2,941 | | | | | | 152 | | |  | | | | | |  | | | | | | |  | | | |  | |  | |
| Sometimes | 4 | 6 | | 2,478 | | 7,793 | | | | | | 799 | | |  | | | | | |  | | | | | | |  | | | |  | |  | |
| Often | 0 | 1 | | 157 | | 786 | | | | | | 528 | | |  | | | | | |  | | | | | | |  | | | |  | |  | |
| Shaded cells depict participants categorised into the same (dark shading) or adjacent (light shading) category at recruitment and at the repeat assessment visit  Percent in same or adjacent category and weighted Kappa given for ordinal variables, percent in same category given for nominal variables | | | | | | | | | | | | | | | | | | | | | | | | | | | | | | | | | | | | | | | | | | | | | | | |
| *This question was only asked to participants who answered 'never' to the questions on how often they consumed processed meat, poultry, beef, lamb, and pork at both the recruitment and the repeat assessment centre visit | | | | | | | | | | | | | | | | | | | | | | | | | | | | | | | | | | | | | | | | | | | | | | | |
| †Those that answered prefer not to answer for this question are excluded | | | | | | | | | | | | | | | | | | | | | | | | | | | | | | | | | | | | | | | | | | | | | | | |
| ‡Spread type is a combination of two variables (n_1428 and n_2654). Participants were coded based on their answers to n_1428, unless they chose do not know or other type of margarine, in which case they were then coded according to their answer to n_2654. Those that answered 'other type of spread/margarine' to n_1428 and then answered 'prefer not to answer' or 'do not know' to n_2654, were coded as 'other type' | | | | | | | | | | | | | | | | | | | | | | | | | | | | | | | | | | | | | | | | | | | | | | | |

| **Supplementary Table S5.** Comparison of the touchscreen estimate of food group intakes with the mean group intakes from the 24-hour dietary assessments | | | |
| --- | --- | --- | --- |
|  | **Reported frequency at recruitment** | **Mean intake (g/d) from the recruitment 24-hour dietary assessment only** | **Mean intake (g/d) from the online 24-hour dietary assessments*** |
| **Intake at recruitment (touchscreen questionnaire)** |  | **n=70,046** | **n=175, 402** |
| Cheese (frequency per week) |  |  |  |
|  | Never | 3 | 5 |
|  | Less than once a week | 9 | 13 |
|  | Once a week | 14 | 16 |
|  | 2-4 times a week | 24 | 23 |
|  | 5-6 times a week | 37 | 33 |
|  | Once or more daily | 49 | 41 |
|  |  |  |  |
| Tea (cups per day) |  |  |  |
|  | 0 | 45 | 90 |
|  | Less than one | 81 | 140 |
|  | 1 | 192 | 244 |
|  | 2 | 356 | 392 |
|  | 3 | 508 | 526 |
|  | 4 | 646 | 636 |
|  | 5 | 784 | 736 |
|  | 6+ | 961 | 881 |
|  |  |  |  |
| Coffee (cups per day) |  |  |  |
| Caffeinated |  |  |  |
|  | Less than one | 60 | 106 |
|  | 1 | 176 | 202 |
|  | 2 | 328 | 323 |
|  | 3 | 470 | 430 |
|  | 4 | 597 | 524 |
|  | 5 | 722 | 618 |
|  | 6+ | 899 | 736 |
|  |  |  |  |
| Decaffeinated |  |  |  |
|  | Less than one | 43 | 59 |
|  | 1 | 137 | 125 |
|  | 2 | 288 | 215 |
|  | 3 | 404 | 305 |
|  | 4 | 533 | 387 |
|  | 5 | 668 | 460 |
|  | 6+ | 786 | 543 |
|  |  |  |  |
| Water (glasses per day) |  |  |  |
|  | 0 | 60 | 131 |
|  | Less than one | 121 | 179 |
|  | 1 | 249 | 280 |
|  | 2 | 449 | 425 |
|  | 3 | 649 | 573 |
|  | 4 | 834 | 697 |
|  | 5 | 1023 | 821 |
|  | 6+ | 1265 | 1016 |
|  |  |  |  |
| Bread (slices per week)* |  |  |  |
| White | 1-5 | 19 | 17 |
|  | 6-10 | 39 | 27 |
|  | 11-15 | 56 | 36 |
|  | 16-20 | 76 | 47 |
|  | 21-25 | 86 | 53 |
|  | 26-30 | 99 | 58 |
|  | 31+ | 130 | 73 |
|  |  |  |  |
| Brown | 1-5 | 7 | 9 |
|  | 6-10 | 17 | 15 |
|  | 11-15 | 26 | 19 |
|  | 16-20 | 39 | 23 |
|  | 21-25 | 47 | 28 |
|  | 26-30 | 50 | 29 |
|  | 31+ | 70 | 44 |
|  |  |  |  |
|  |  |  |  |
| Wholemeal or wholegrain |  |  |  |
|  | 1-5 | 10 | 12 |
|  | 6-10 | 22 | 18 |
|  | 11-15 | 33 | 25 |
|  | 16-20 | 45 | 33 |
|  | 21-25 | 57 | 39 |
|  | 26-30 | 69 | 48 |
|  | 31+ | 94 | 68 |
|  |  |  |  |
| Breakfast cereal (bowls per week) |  |  |  |
| Bran cereal | 1 | 4 | 4 |
|  | 2 | 7 | 5 |
|  | 3 | 12 | 6 |
|  | 4 | 16 | 8 |
|  | 5 | 23 | 11 |
|  | 6 | 28 | 13 |
|  | 7 | 31 | 19 |
|  | 8+ | 37 | 21 |
|  |  |  |  |
|  |  |  |  |
|  |  |  |  |
| Biscuit cereal | 1 | 4 | 5 |
|  | 2 | 9 | 7 |
|  | 3 | 14 | 8 |
|  | 4 | 20 | 10 |
|  | 5 | 27 | 14 |
|  | 6 | 31 | 18 |
|  | 7 | 36 | 24 |
|  | 8+ | 44 | 30 |
|  |  |  |  |
|  |  |  |  |
|  |  |  |  |
| Oat cereal | 1 | 30 | 27 |
|  | 2 | 42 | 34 |
|  | 3 | 59 | 40 |
|  | 4 | 83 | 51 |
|  | 5 | 110 | 65 |
|  | 6 | 129 | 84 |
|  | 7 | 146 | 107 |
|  | 8+ | 173 | 129 |
|  |  |  |  |
|  |  |  |  |
|  |  |  |  |
| Muesli | 1 | 8 | 11 |
|  | 2 | 15 | 14 |
|  | 3 | 28 | 19 |
|  | 4 | 39 | 22 |
|  | 5 | 53 | 31 |
|  | 6 | 61 | 39 |
|  | 7 | 72 | 50 |
|  | 8+ | 85 | 56 |
|  |  |  |  |
|  |  |  |  |
|  |  |  |  |
| Other (e.g. Cornflakes, Frosties) | 1 | 4 | 5 |
|  | 2 | 6 | 7 |
|  | 3 | 12 | 9 |
|  | 4 | 17 | 10 |
|  | 5 | 22 | 13 |
|  | 6 | 25 | 17 |
|  | 7 | 28 | 20 |
|  | 8+ | 36 | 24 |
| *Estimated usual intake from the mean group intakes from the 24-hour dietary assessments completed online | | | |

**Supplementary References**

6. Ministry of Agriculture, Fisheries and Food (1993) *Food Portion Sizes*. London: HMSO.

7. Finglas PM, Roe MA, Pinchen HM, *et al.* (2015) *McCance and Widdowson’s The Compostion of Foods, Seventh Summary Edition*. Cambridge: Royal Society of Chemistry.
